# A Review on Cs-Based Pb-Free Double Halide Perovskites: From Theoretical and Experimental Studies to Doping and Applications

**DOI:** 10.3390/molecules26072010

**Published:** 2021-04-01

**Authors:** Fatemeh Heidari Gourji, Dhayalan Velauthapillai

**Affiliations:** Department of Computer Science, Electrical Engineering and Mathematical Sciences, Western Norway University of Applied Sciences, Inndalsveien 28, 5063 Bergen, Norway

**Keywords:** Cs-based Pb-free double halide perovskites, applications of double halide perovskites, doping

## Abstract

Despite the progressive enhancement in the flexibility of Pb-based perovskites for optoelectronic applications, regrettably, they are facing two main challenges; (1) instability, which originates from using organic components in the perovskite structure, and (2) toxicity due to Pb. Therefore, new, stable non-toxic perovskite materials are demanded to overcome these drawbacks. The research community has been working on a wide variety of Pb-free perovskites with different molecular formulas and dimensionality. A variety of Pb-free halide double perovskites have been widely explored by different research groups in search for stable, non-toxic double perovskite material. Especially, Cs-based Pb-free halide double perovskite has been in focus recently. Herein, we present a review of theoretical and experimental research on Cs-based Pb-free double halide perovskites of structural formulas Cs_2_M^+^M^3+^X6 (M^+^ = Ag^+^, Na^+^, In^+^ etc.; M^3+^= Bi^3+^, In^3+^, Sb^3+^; X = Cl^−^, Br^−^, I¯) and Cs_2_M^4+^X_6_ (M^4+^ = Ti^4+^, Sn^4+^, Au^4+^ etc.). We also present the challenges faced by these perovskite compounds and their current applications especially in photovoltaics alongside the effect of metal dopants on their performance.

## 1. Introduction

During the last ten years, Pb-based perovskites as a promising subcategory of solar cell materials have attracted intense attention of the research community due to their unique advantages. In 2009, for the first time, Miyasaka et al. published a paper demonstrating the use of methylammonium lead triiodide (CH_3_NH_3_PbI_3_) as a light absorber instead of dye in dye sensitized solar cells (DSSCs) with an efficiency of 3.8% [1]. A few years after, a solid hole transporting layer named Spiro-MeOTAD replaced the liquid electrolyte in these structures which helped to increase the efficiency of perovskite solar cells (PSCs) to 9.7% [2]. Since then, the number of research articles in this field exponentially increased, and considerable advances in power conversion efficiency exceeding 25.2% are reported for PSCs [3].

The general formula of perovskite is ABX_3_ where A is a monovalent cation (methylammonium (MA^+^), formamidinium (FA^+^), or Cs^+^), B is a divalent metal (Pb^2+^) and X is halide anion (I^−^, Br^−^, and Cl^−^) [4]. Depending on A-cation, perovskites can be categorized into (1) organic-inorganic hybrid or (2) all-inorganic halide perovskite [5]. Possession of unique advantages including high extinction coefficient [6,7], low exciton binding energy [8], and tunable bandgap [9], turn the metal halide perovskites as superb carrier transporters and photo absorbers. It is noteworthy that these materials are also used not only in PSCs, but also in other optoelectronic devices, such as light-emitting diodes (LEDs) [10], lasers [11], photodetectors [12,13], and X-ray detectors [14].

However, there are few drawbacks regarding Pb-based halide perovskites as an active layer in PV applications, which must be tackled in order to achieve full scale application [15]. The first concern is of lead in perovskite materials as Pb is a well-known heavy metal [16]. Exposure to lead causes a detrimental effect on humans and surprisingly the absorptions of this metal in children’s body is four to five times larger than an adult’s human body [17]. Acute exposure to Pb can result in kidney damages, brain damages, and gastrointestinal disease, and chronic exposure to lead may induce adverse effects on the blood pressure, kidneys, central nervous system, and vitamin D metabolism [18,19]. Pb also can replace Calcium and interact with proteins and, as a result, disrupts the neurotransmitter release and bone mineral density [17,19]. In order to decrease the usage amount of hazardous materials, including Pb in electronic and electric devices, different restrictions of hazardous substances (RoHS) policies were implemented in many countries with the aim of reducing the risk to both human health and environment [17,20].

The second challenge with organic-inorganic PSCs is the weak operational stability of organic lead halide perovskite due to organic formamidinium (FA^+^) or methylammonium (MA^+^) which make them vulnerable to degradation upon exposure to light, high temperature and humidity [21,22].

Accordingly, in order to achieve more stable and non/less toxic PSCs, different strategies have been adopted including (1) utilization of a protective layer around perovskite material as well as resilience development of perovskite layer [23], (2) using mixed halide anions and mixed A- cations [24,25], (3) applying of additives and doping agents [25,26,27] such as monovalent (Cu^+^ [28], Ag^+^ [28,29], Na^+^ [28,30], etc.), divalent (Mn^2+^ [30], Sr^2+^[31], Zn^2+^ [32], etc.) and trivalent metal ions (Bi^3+^ [33] and Sb^3+^ [34]), and (4) synthesis of 2D halide perovskites [35,36,37]. However, these approaches were not able to overcome the aforementioned challenges adequately. Therefore, new designs of perovskite materials, applicable to all optoelectronic devices, have been suggested through theoretical and experimental studies by different research groups where Pb^2+^ and MA^+^/FA^+^ were replaced by less/non-toxic inorganic components.

In a number of studies, it has been shown that incorporation of Cs^+^ instead of applying MA^+^/FA^+^ in perovskite structure has led to a broad band of advantages, including decreasing crystallization temperature, enhancing the absorption before and after annealing, decreasing the side phases, improving photogenerated carrier lifetime, and reducing the density of trap states, etc., which has resulted in higher PCEs for the PSCs and has also led to better stability of perovskite structure [38]. In order to tackle the toxicity issue, Sn^2+^ and Ge^2+^ ions have been suggested as the first logical substitution of Pb^2+^ in perovskite structure since these metal ions have similar electronic configuration of *s^2^p^2^* and thereby similar chemical properties [39,40,41]. It is shown that Sn-based perovskites provide numerous advantages such as narrower bandgap [42], higher charge carrier mobilities [42], and longer diffusion length [43] compared to their Pb analogs. But the idea of using these ions did not meet the expected results because Sn^2+^ and Ge^2+^ ions tend to be oxidized to their stable oxidations state of 4+ [44]. Bi^3+^ and Sb^3+^ with similar isoelectronic structure were the other explored alternatives to Pb^2+^ ion [41,45]. However, their chemical bonding preference and their 3+ oxidation state make them suitable for fitting in tetragonal structure in A_3_M_2_I_9_ perovskite, meaning that they have a low-dimensional structure (2D) causing poor performance [5,45,46,47].

Double perovskite crystals with a general formula A_2_M^+^M^3+^X_6_ and A_2_M^4+^X_6_ (where M is the metal ion) are the other alternative and promising lead-free perovskite materials, that have been proposed for photovoltaic and other optoelectronic applications [48,49,50]. In this structure, two different metal ions with different oxidation states of 1+ and 3+, are substituted by two divalent Pb^2+^ metal ion in a three-dimensional perovskite structure. The space group of this lead-free perovskite materials is *Fm3*$\overset{¯}{m}$ with two different octahedra groups comprised of M^+^ and M^3+^ in a rock salt face-centered cubic structure [50].

This review aims to focus on experimental and theoretical studies of Cs-based inorganic Pb-free double halide perovskite materials in the form of Cs_2_M^+^M^3+^X6 (M^+^ = Ag^+^, Na^+^, In^+^ etc.; M^3+^ = Bi^3+^, In^3+^, Sb^3+^; X = Cl^−^, Br^−^) and Cs_2_M^4+^X_6_ (M^4+^ = Ti^4+^, Sn^4+^, Au^4+^ etc.) alongside their results and applications in different optoelectronic devices, including solar cells, light-emitting diodes (LEDs), X-ray detectors, and photodetectors. We also present the effect of elemental substitution on the performances of these materials.

## 2. Double Halide Perovskites

The two key features of Pb-based halide perovskites (ABX_3_) which result in excellent optoelectronic properties are high symmetry of BX_6_ octahedral and the unique electronic configuration of Pb^2+^ (6s^2^ 6p^0^) which leads to strong Pb 6s- I 5p antibonding coupling [51]. Therefore, to obtain a promising Pb-free solar absorber candidate in perovskite structure, the symmetry of octahedra should be preserved. Octahedral rotation distortion, like twisting or tilting, may affect the symmetry properties of target crystals. In order to maintain high stability and high symmetry of cubic structure, two different parameters named Goldschmidt’s tolerance factor (t = R_A_+R_X_)/(√2(R_B_+R_X_)), and octahedral factor (μ = R_B_/Rx) should be considered. For ideal perovskite structure “t” is between 0.8 and 1 and “μ” is larger than 0.414 [52].

Meanwhile, some research groups have considered other factors for designing efficient Pb-free double halide perovskites for solar cell applications. Meng et al. [53], by carrying out a computational study suggested that inversion symmetry-induced parity-forbidden transitions should be considered for designing new double halide perovskites. In their study, they indicated that for most double halide perovskites with a direct bandgap like Cs_2_AgInCl_6_, transition from valance band maximum (VBM) to conduction band minimum (CBM) is forbidden because of inversion symmetry in their structure. And this is likely one of the reasons for their poor photovoltaic performance [53]. Xiao et al. [51] demonstrated that for gaining optimal and efficient lead-free halide perovskites for photovoltaic performance, besides structural dimensionality, electronic dimensionality should also be taken into account. This new concept explains that connectivity of electronic orbitals, which form the conduction band minimum (CBM) and valance band maximum (VBM), is another essential parameter for understanding the photovoltaic properties of all reported perovskites. Perovskites with three-dimensional (3D) crystal and electronic structures are the most desired for good photovoltaic performance [51].

**Figure 1:**
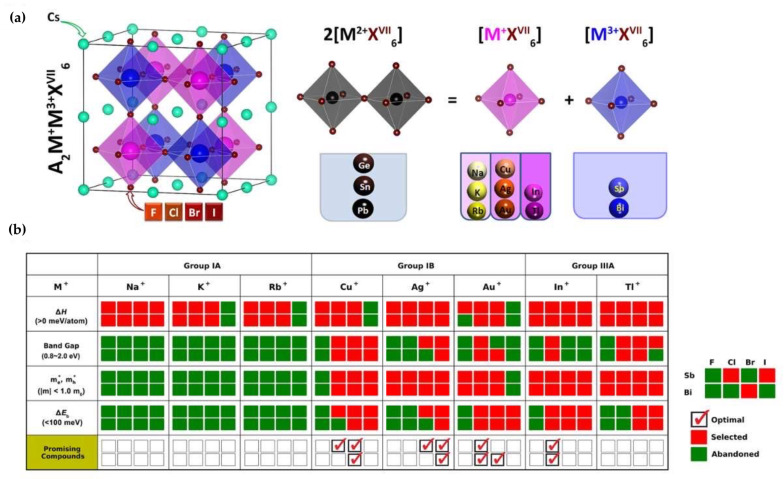
(**a**) Suggested structure of double halide perovskite with a formula of A_2_B^+^B^3+^X_6_ using the idea of transmutation and; (**b**) materials screening process considering the decomposition enthalpy (∆H), bandgap (E_g_), carrier effective masses (m_e_* and m_h_*) and exciton binding energy (∆E). Reproduced with permission from [54]. Copyright 2017, American Chemical Society.

Considering all important and affecting parameters, Zhao et al. [54], through the first-principals calculation exploited the cation transmutation idea to explore all-inorganic Pb-free double halide perovskites for photovoltaic application by replacing Pb^2+^ by Bi^3+^ or In^3+^. Among 64 different potential considered candidates, only 11 compounds with intrinsic thermodynamic stability, suitable band gaps, small carrier effective masses, and low exciton binding energies have been identified as promising absorbers. Figure 1a,b show the screening process of the materials based on properties like decomposition enthalpy (∆H), carrier effective masses (m_e_* and m_h_*) and exciton binding energy (∆E_b_) [54]. In the following, we will present the successfully investigated Cs-based Pb-free all-inorganic double halide perovskites with their current potential applications in various optoelectronic devices, along with the effect of elemental substitution on their optical and structural characterizes.

### 2.1. Cs/Bi^3+^-Based Double Halide Perovskites

For many decades, Bi (bismuth) has been applied as an eco-friendly and non-toxic metal with interesting properties in diverse applications. Bi has been introduced as a suitable replacement for Pb because of comparable density and similar electronic configuration [55,56]. Due to the significant features, Bi has been one of the first examined candidates for developing Pb-free double halide perovskite materials for PV applications [48,49,50]. This idea was developed by three independent research groups which simultaneously, published their successful investigations by replacing Pb^2+^ with heterovalent substitution of Bi^3+^ and Ag^+^ to form a lead-free double halide perovskite in 2016 [48,49,50].

For the sake of completeness, we have provided a list of Cs/Bi^3+^-based halide double perovskites along with their theoretical and experimental bandgap values and other information including morphology and applied synthetic methods in Table 1. In order to get a good overview, we present the results from theoretical and experimental studies separately.

#### 2.1.1. Cs_2_M^+^Bi^3+^X_6_: Theoretical Studies

**Figure 2:**
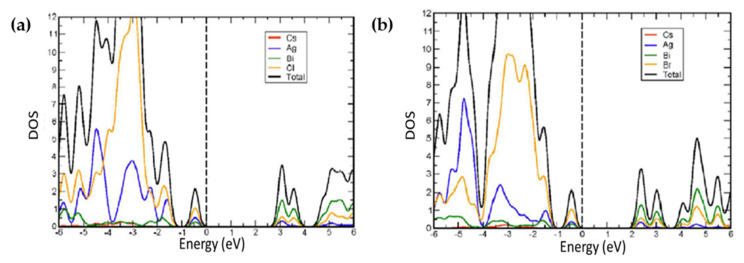
Atomic partial density of states for; (**a**) Cs_2_AgBrBr_6_ and; (**b**) Cs_2_AgBrCl_6_. Reproduced with permission from [49]. Copyright 2016, American Chemical Society.

McClure et al. [49] studied Cs_2_AgBiBr_6_ and Cs_2_AgBiCl_6_ as the two first potential double halide perovskite compounds, by employing DFT analysis on VASP simulation software. The indirect bandgap of 2.06 eV for Cs_2_AgBiBr_6_ and 2.62 eV for Cs_2_AgBiCl_6_ were obtained as presented in Table 1. According to the atomic partial density of states, the indirect bandgap resulted from the combination of Ag 4d orbitals with halogens 3p/4p orbitals which led to sufficient changes in the valence band as shown in Figure 2a,b. The study has also shown that hole effective masses were lighter (Cs_2_AgBiCl_6_ = 0.15 m_h_; Cs_2_AgBiBr_6_ = 0.14 m_h_) than their CsPbX_3_ analogs (CsPbCl_3_ = 0.35 m_h_; CsPbBr_3_ = 0.37 m_h_) and the electron effective masses were also comparable (Cs_2_AgBiCl_6_ = 0.53 m_e_; Cs_2_AgBiBr_6_ = 0.37 m_e_) with CsPbX_3_ compounds (CsPbCl_3_ = 0.41 m_e_; CsPbBr_3_ = 0.34 m_e_).

Volonakis et al. [50] selected a different range of metals comprised of pnictogen (Bi^3+^, Sb^3+^ (group VA of periodic table)) and noble metals (Ag^+^, Au^+^, Cu^+^) for computational and experimental studies. By using density functional theory in the local density approximation (DFT-LDA), they found all these compounds to have indirect bandgaps of less than 2.7 eV. By changing both halides from I^−^ to Cl^−^ and the pnictogen from Bi^3+^ to Sb^3+^, the bandgap was seen to be increasing. However, changing of the noble metals did not have any effect on this trend, since CBM and VBM mostly have pnictogen-p and halogen-p character, respectively. This means that by moving up in halogen (from I^−^ to Cl^−^) and pnictogen group (from Bi^3+^ to Sb^3+^) in the periodic table, their p-state energy will be decreased, and consequently both VBM and CBM will be lowered. Another notable examined parameter was the smaller carrier effective masses in these compounds (between 0.1 and 0.4) compared to relevant MAPbI_3_.

**Figure 3:**
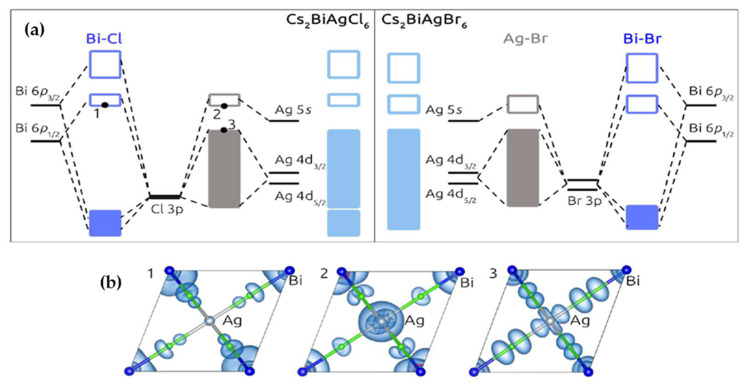
(**a**) Comparison between the molecular orbitals diagram of Cs_2_AgBrCl_6_ and Cs_2_AgBrBr_6_ and; (**b**) Square modulus of the wave functions for states marked from **1** to **3** on the molecular orbital diagram of Cs_2_BiAgCl_6_. **1** represents the Bi-6p_1/2_/halide-p antibonding orbitals at the bottom of the conduction band (at Γpoint). **2** is the antibonding Ag-5s/halide-p at the L-point of the conduction band), while **3** corresponds to the Ag-4d/halide-p antibonding orbitals found at the top of the valence band (at X-point). Reproduced with permission from [57]. Copyright 2016, American Chemical Society.

Filip et al. [57] focused on the electronic properties of Cs_2_AgBiX_6_ (X = Cl¯and Br^−^) compounds through theoretical calculations. To obtain accurate bandgap values, they used the experimental crystal structures data within the DFT-LDA calculation in the quantum espresso suit with and without spin-orbit coupling. Based on obtained molecular orbitals diagram through the atom-projected density of states, they indicated that because of smaller energy difference in Ag-d and Br-p states in comparison with Ag-d and Cl-p, valence band width was reduced. Moreover, the delocalized nature of Br-4p orbitals resulted in more overlaps with Bi-6p, and thereby the width of conduction band increased as illustrated in Figure 3. Study of quasiparticle calculation for determination of the bandgap (1.8 eV for Cs_2_AgBiBr_6_ and 2.4 eV for Cs_2_AgBiCl_6_) showed a good agreement with their experimental findings (1.9 eV for Cs_2_AgBiBr_6_ and 2.2 eV for Cs_2_AgBiCl_6_) [57].

Xiao et al. [69] investigated the thermodynamic stability of Cs_2_AgBiBr_6_ by DFT calculation, and claimed that Ag vacancies were shallow accepters that resulted in intrinsic p-type conductivity in Cs_2_AgBiBr_6_. On the other hand, the existence of some dominant deep defects such as Bi vacancies (V_Bi_) and AgBi antisites give rise to poor photovoltaic performance. Accordingly, Xiao et al. [69], in order to reduce the formation of deep defects, suggested that the synthesis of material preferably should be done under Bi-poor/Bi-rich growth conditions. 

Xiao et al. [67] studied Cs_2_In^+^M^3+^X_6_ (M = Bi^3+^, and X = halogens) double halide perovskites, both theoretically and experimentally. Their results indicated that high-energy-laying in 5s^2^ state of In^+^ is substantially responsible for promising photovoltaic performance. However, due to the oxidation of In^+^ to In^3+^, and the reduction of Bi^3+^ to its metal form, the whole perovskite structure becomes unstable.

Since optimizing the bandgap is an important factor for enhancing the efficiency of PSCs, Yang et al. [58] proposed by changing the atomic arrangement in Cs_2_AgBiBr_6_ crystal structure, the bandgap can be narrowed. Using Monte Carlo and DFT calculations performed in VASP code by employing HSE06 with SOC, they showed that increasing the temperature up to 1200 K would increase the energy and consequently, phase transitions would occur. In this condition, Ag^+^ and Bi^3+^ ions randomly occupy the M-site in A_2_M^+^M^3+^X_6_ structure, and this leads to reduction of bandgap from 1.93 eV to pseudo-direct bandgap of 0.44 eV.

#### 2.1.2. Cs_2_M^+^Bi^3+^X_6_: Experimental Studies

In this section, we provide detailed synthesis methods of Cs/Bi^3+^-based double halide perovskites and their characterization results. This section based on obtained morphologies is divided into two categories, namely, crystalline and thin-film structures in 1.2.1 and 1.2.2, respectively. This detailed information of the experimental studies is also summarized in Table 1.

- Cs_2_M^+^Bi^3+^X_6_: Single-Crystals, Polycrystalline and Nanocrystals-Based Perovskites

**Figure 4:**
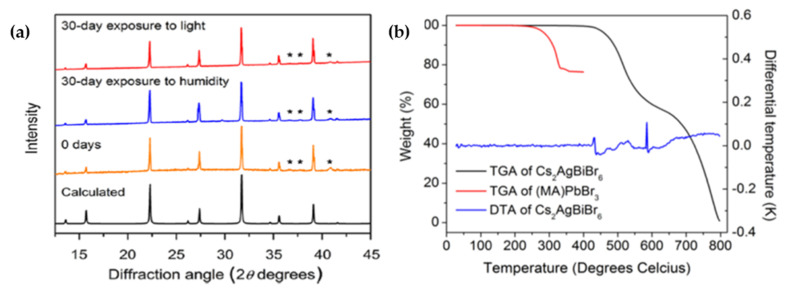
(**a**) PXRD pattern of Cs_2_AgBiBr_6_ after exposure to humidity (55% RH) and light (0.75 Sun), * and ** refers to the signals from sample holder; (**b**) Thermogravimetric analyses of Cs_2_AgBiBr_6_ and (MA)PbBr_3_ at a scan rate of 5 °C/min and 1 °C/min, respectively. Solid (MA)PbBr_3_ shows an initial mass loss at 176 °C. Solid Cs_2_AgBiBr_6_ shows an initial mass loss at 430 °C. DTA of Cs_2_AgBiBr_6_ shows no phase changes until the mass loss onset. Reproduced with permission from [48]. Copyright 2016, American Chemical Society.

Slaveney et al. [48] synthesized double halide perovskite Cs_2_AgBiBr_6_ with photoluminescence (PL) lifetime of approximately 660 ns with an indirect bandgap of 1.95 eV. Compared to MAPbI_3_, this compound showed higher stability against moisture and heat and had long room-temperature PL lifetime (736 ns to 1 μs for MAPbI_3_ films) which is a good characteristic for photovoltaic performance. Interestingly, the obtained PXRD (Powder X-ray diffraction) pattern did not show any evidence of material decompaction as shown in Figure 4a. Thermogravimetric analysis (TGA) showed that the material was stable up to 430° which could be due to the replacement of unstable organic cation MA^+^ with Cs^+^ ions as depicted in Figure 4b. 

**Figure 5:**
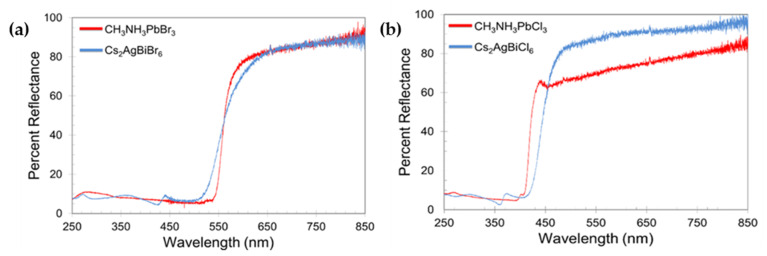
Diffuse reflectance spectra of; (**a**) MAPbBr_3_ and Cs_2_AgBrBr_6_; (**b**) MAPbCl_3_ and Cs_2_AgBrCl_6_. Reproduced with permission from [49]. Copyright 2016, American Chemical Society.

McClure et al. [49] prepared polycrystalline Cs_2_AgBiBr_6_ and Cs_2_AgBiCl_6_ by applying solid state and solution-based methods. The PXRD analysis confirmed the 3D structure of these compounds in corner connected octahedra with alternating Ag^+^ and Bi^3+^ in a rock salt ordering. However, according to PXRD and Rietveld refinements, due to large number of displacement parameters (Beq) and low bond valence sum of Cesium ion, phase transition could be expected below room temperature. UV-Vis diffuse reflectance spectra showed significant similarities between Cs_2_AgBiX_6_ and Pb-based analogs (CH_3_NH_3_PbX_3_) although there were some minor differences at photon energies above the absorption onset as shown in Figure 5a,b. Using Kubelka-Munk equation and Tauc plot, the optical indirect bandgap of 2.19 eV and 2.77 eV were obtained for Cs_2_AgBiBr_6_ and Cs_2_AgBiCl_6_, respectively which were close to their theoretically calculated values (2.06 eV for Cs_2_AgBiBr_6_ and 2.62 eV for Cs_2_AgBiCl_6_) [46]. In order to investigate the light and chemical stability, samples were placed in both dark and light conditions under ambient environment for one month. The obtained UV-Vis diffuse reflectance spectra analysis of the samples after 14 and 28 days exhibited that Cs_2_AgBiBr_6_ was less light stable than Cs_2_AgBiCl_6_ and the conclusion was that encapsulation of the structures might be needed for PV application.

Volonakis et al. [50] successfully prepared single-phase Cs_2_AgBiCl_6_ samples through the solid-state reaction in a sealed fused silica ampoule. The structural and optical measurements by using single-crystal X-ray diffraction (SCXRD), UV-Vis spectroscopy and photoluminescence showed that the Cs_2_AgBiCl_6_ perovskite belongs to *Fm3*$\overset{¯}{m}$ space group while BiCl_6_ and AgCl_6_ octahedra are alternately placed in a rock-salt face-centered cubic structure with an indirect bandgap of 2.2 eV.

It is noticeable that the three abovementioned articles [48,49,50] presented different experimental bandgap values for Cs_2_AgBiCl_6_ (2.2 eV to 2.7 eV) and Cs_2_AgBiBr_6_ (1.83 eV to 2.19 eV) which is mainly due to the different synthesis methods (solid state preparation and solution processing) as well as utilizing different measurement techniques and methods such as UV-Vis spectroscopy and UV-Vis diffuse reflectance, Tauc plot, etc. for calculating bandgaps [57]. To overcome these discrepancies, Filip et al. [57] by employing the same solid state and solution-based synthesis methods prepared stable Cs_2_AgBiBr_6_ and Cs_2_AgBiCl_6_ single crystals under ambient environment. Through the optical measurements including UV-Vis spectroscopy and PL, they obtained an indirect bandgap of 1.9 eV and 2.2 eV for Cs_2_AgBiBr_6_ and Cs_2_AgBiCl_6_, respectively.

**Figure 6:**
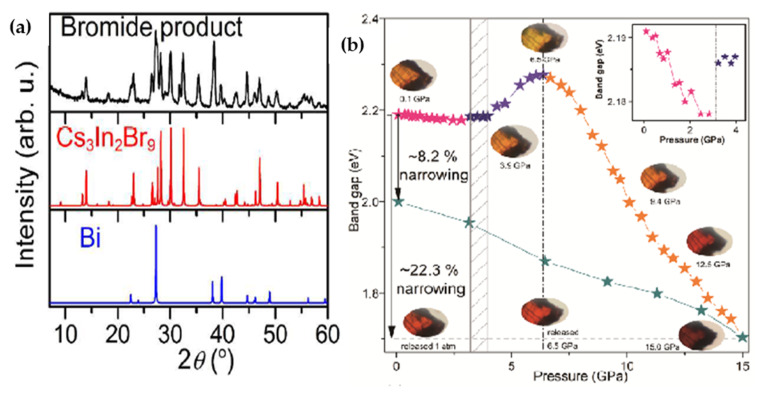
(**a**) PXRD pattern of the bromide product and simulated PXRD pattern for Cs_3_In_2_Br_9_ and Bi. Reproduced with permission [67]. Copyright 2017, American Chemical Society; (**b**) evolution of bandgap in Cs_2_AgBrBr_6_ structure at high pressure with representative optical micrographs indicating piezochromic transitions. Reproduced with permission from [56]. Copyright 2017, Wiley-VCH.

Xiao et al. [67] used In^+^ instead of Ag^+^ and synthesized Cs_2_In^+^Bi^3+^X_6_ (X = halogens) double halide perovskites using solid-state reaction method. Their experimental investigation using SCXRD (Single Crystal X-ray diffraction) and PXRD exhibited that In^+^-based halide double perovskites spontaneously decomposes into In^3+^-based ternary materials such as CsInI_4_, Cs_3_In_2_Br_9_ and Cs_3_In_2_Cl_9_ (Figure 6a). In fact, In^1+^ is an unstable and unusual oxidation state in inorganic solids and there are only a few reported double perovskite compounds based on In^+^ [70,71].

Li et al. [56] by applying the high-pressure method, which previously had been used for size-dependent phase transformation [72] indicated that the bandgap of Cs_2_AgBiBr_6_ through high-pressure method was reduced by 22.3% from 2.2 eV to 1.7 eV. Using UV-vis spectroscopy as optical measurement, they observed by increasing the pressure to ~15 GPa, the color of the product changed to brown black and absorption peak exhibited a continues redshift. Interestingly, even after releasing the pressure, the bandgap value was observed to be around ≈2.0 eV (Figure 6b).

The angle dispersive X-ray diffraction (ADXRD) analysis of the sample illustrated that by employing a wide range of pressure, a minimized octahedral tilting through *ab* plane occurs, and this decreased the Bi-Br-Ag bond angle from 180° to 166.4°. It is believed that this is one of the major factors of narrowing the bandgap of Cs_2_AgBiBr_6_. 

In 2018, Hoye et al. [60] explored the carrier lifetime and recombination mechanism in the Cs_2_AgBiBr_6_ thin film in more detail. Both single crystals and thin film of Cs_2_AgBiBr_6_ were prepared through the slow precipitation method of saturated solution at low temperature. Based on optical measurements, the fundamental carrier lifetime for Cs_2_AgBiBr_6_ thin film was shown to be 1.4 μs. The carrier density of Cs_2_AgBiBr_6_ was calculated to be 2.2 × 1016 cm^−3^ under 1 sun, which was larger than MAPbI_3_ steady-state carrier density (5.2 × 1015 cm^−3^ under 1 sun). This clearly indicates the benefit of utilizing Cs_2_AgBiBr_6_ in solar cells.

**Figure 7:**
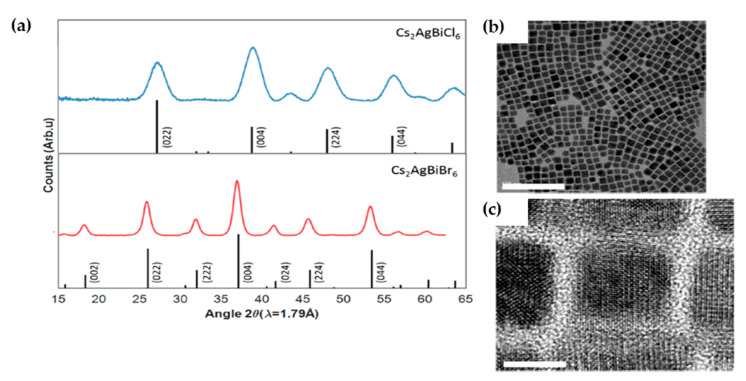
(**a**) XRD pattern of Cs_2_AgBiBr_6_ (red) and Cs_2_AgBiCl_6_ (blue) NCs in comparison with reported XRD patterns where scale bars refer to the other published XRD patterns for Cs_2_AgBiBr_6_ and Cs_2_AgBiBr_6_ perovskites; (**b**) low-resolution and; (**c**) high-resolution TEM images of Cs_2_AgBiBr_6_ NCs showing atomic lattice fringes. Reproduced with permission from [59]. Copyright 2018, American Chemical society.

Bekenstein et al. [59] selected hot-injection method for preparation of Cs_2_AgBiBr_6_ nanocrystals which previously was employed for synthesis of CsPbX_3_ and CsPbCl_3_ NCs by Protesescu et al. [73], and Xu et al. [74], respectively. The XRD and HRTEM analysis indicated that the crystals had the cubic *Fm3*$\overset{¯}{m}$ structure with the side length of 8–15 nm as shown in Figure 7a–c.

The color of the sample was distinctly yellow, which was different from the previously prepared samples’ color based on solid-state and solution-processed methods (orange-red) [48,49,50]. The stability of the NCs was examined through the changing of solvents and changing of ligands. Solvents with higher polarity caused less stable NCs and by introducing the excess amount of primary and tertiary amines, absorption and emission features of the sample disappeared, which suggested that Cs_2_AgBiBr_6_ and CsPbI_3_ had similar surface chemistry. Furthermore, it was demonstrated [59], due to the Ag diffusion, reduction and coalescence, Cs_2_AgBiBr_6_ NCs in solution were structurally unstable and degraded to Cs_3_Bi_2_Br_9_ and Cs_3_BiBr_6_ byproduct NCs. However, by controlling the solvents evaporation rate, less degradation was occurred on NCs superlattices assembled into the ordered NC solids including strong ligand-ligand interaction.

**Figure 8:**
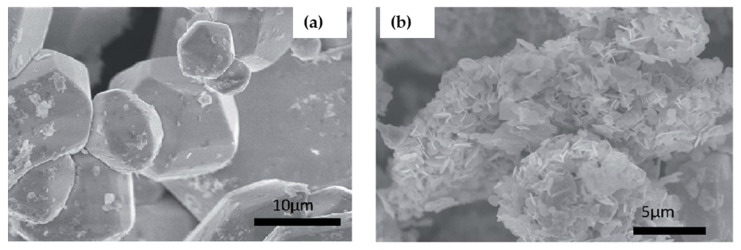
(**a**) multiple shuttle-like crystals from high acidity synthesis and; (**b**) clusters of nano-plates from non-acid synthesis. Reproduced with permission from [75]. Copyright 2018, Royal Society of Chemistry.

Zhang et al. [75], as presented in Table 2, replaced Ag^+^ in Bi-based halide double perovskite with Na^+^ ion for fabricating the solar cell, and Cs_2_NaBiI_6_ perovskite was synthesized by facile one-step hydrothermal process. The material showed tolerance factor of 0.849 and octahedral factor of 0.466 with bandgap of 1.66 eV. The SEM images showed by increasing the concentration of solvent (HI), up to 6 M, the crystal growth got improved as shown in Figure 8a,b.

In 2019, Zelewski et al. [61] by combination of photoluminescence excitation (PLE) and Raman spectroscopy demonstrated that the PL emission in Cs_2_AgBiBr_6_, was dominated by the strong electron-phonon coupling due to the relatively large Huang-Rhys factor (S = 11.7). It was also revealed that the excitation resonant of the color center in ionic crystals of Cs_2_AgBiBr_6_ was more responsible for the PL emission rather than band to band transition. It was also proven that the color centers of ionic crystals were coupled with crystal lattice vibrations [76]. Local lattice deformation would occur if any of these centers were occupied. This led to energy offset in color center between ground state (unoccupied) and excited states (occupied) [61].

- Cs_2_M^+^Bi^3+^X_6_: Film-Based Perovskites

In following section, we present the studies in which Cs_2_M^+^Bi^3+^X_6_ perovskites prepared as thin film for PV application and the results are presented in Table 2.

**Figure 9:**
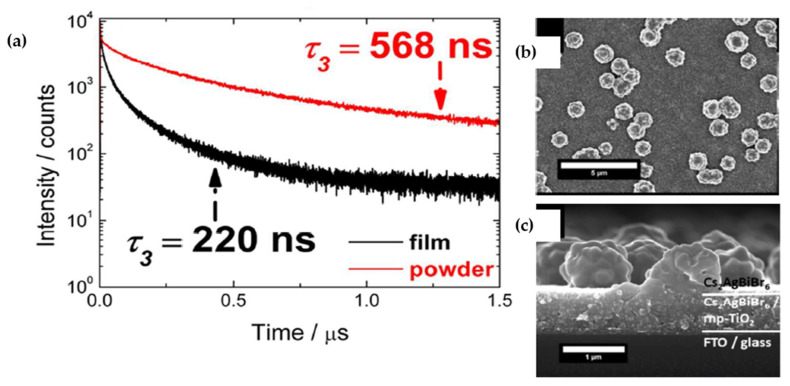
(**a**) TCSPC decays of a Cs_2_AgBiBr_6_ film on glass and polycrystalline powder; (**b**) SEM top-view and; (**c**) SEM cross-section images of the Cs_2_AgBiBr_6_ film prepared with a 75 °C preheating step on mp-TiO_2_. Reproduced with permission from [77]. Copyright 2017, Royal Society of Chemistry.

Although other research groups were employing halide acids as solvent, in 2017, Greul et al. [77] for the first time used dimethyl sulfoxide (DMSO) as the solvent in the synthesis of Cs_2_AgBiBr_6_ film. By annealing at temperatures higher than 250° all the side phases such as AgBr and Cs_3_Bi_2_Br_9_ were removed. They reported that adopting a preheating step at 75° right before the spin coating of Cs_2_AgBiBr_6_ film led to enhanced surface coverage and increased optical absorption. However, the Cs_2_AgBiBr_6_ film exhibited faster photoluminescence decay time (220 ns) in comparison with polycrystalline powders (568 ns) due to their large concentration of trap states (Figure 9a). Also, SEM images of the film indicated some agglomeration on the surface of spin coated film which contributed to the fast crystallization process (Figure 9b,c).

**Figure 10:**
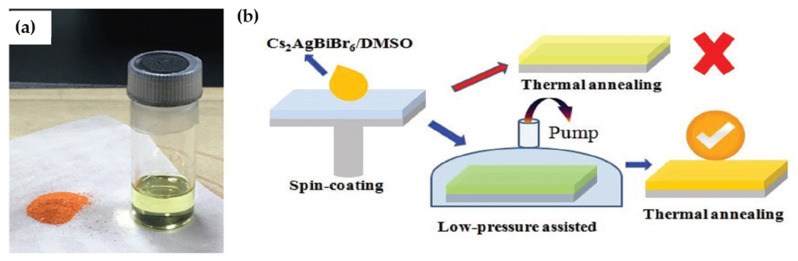
(**a**) images of Cs_2_AgBiBr_6_ powder and Cs_2_AgBiBr_6_ solution in DMSO and; (**b**) the film fabrication process diagram. Reproduced with permission from [78]. Copyright 2018, Wiley-VCH.

Wu et al. [78] employed low-pressure assisted (LPA) method for preparation of Cs_2_AgBiBr_6_ film. In this method, as-synthesized Cs_2_AgBiBr_6_ powder [48] was dissolved in DMSO and then spin-coated on an ITO/glass substrate. The prepared film then was quickly moved to a low-pressure chamber (20 Pa) and finally was annealed thermally (Figure 10a,b). Using the LPA method led to enhanced crystallinity and grain size.

**Figure 11:**
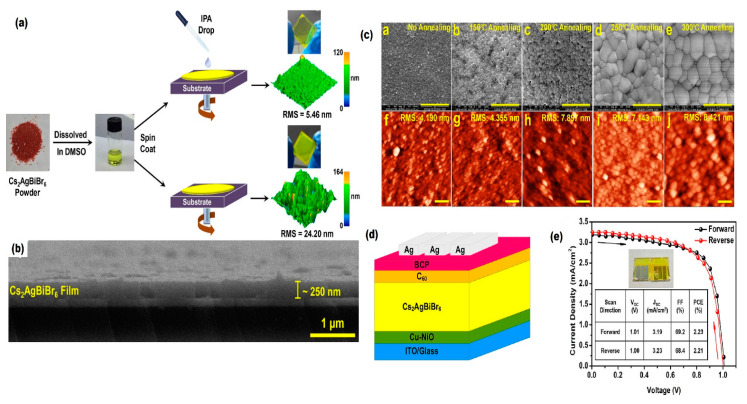
(**a**) Schematic illustration of the spin-coating process (with and without anti-solvent dropping protocol); (**b**) Cross-sectional SEM image of a Cs_2_AgBiBr_6_ film obtained by IPA dipping; (**c**) SEM images and AFM images of Cs_2_AgBiBr_6_ films annealed at different temperatures; (**d**) Structure of the inverted PSC devices fabricated in this work and; (**e**) J-V curves of the best-performing device based on Cs_2_AgBiBr_6_ films obtained at different fabrication conditions. Reproduced with permission from [81]. Copyright 2018, Wiley-VCH.

Gao et al. [81] inspired by the LPA synthesis method [88], adopted an anti-solvent dropping methodology for synthesis of Cs_2_AgBiBr_6_ film as depicted in Figure 11a,b. Combination of this method with post-annealing at high temperature resulted in smooth morphology, micro-sized grains and high crystallinity (Figure 11c).

**Figure 12:**
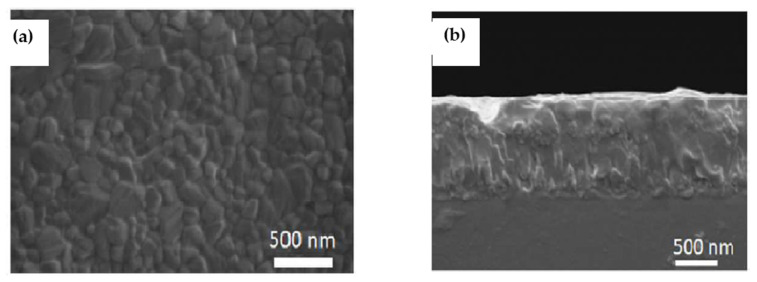
(**a**) Top-view SEM image of obtained Cs_2_AgBiBr_6_ film by spin coating at room temperature using the anti-solvent treatment with chlorobenzene; (**b**) cross-section SEM image of FTO/c-TiO_2_/m-TiO_2_/double perovskite sample, upon annealing at 280 °C. Reproduced with permission from [80]. Copyright 2018, American Chemical Society.

Pantaler et al. [80] used chlorobenzene as anti-solvent before the annealing step during the film formation to obtain the smooth layer without any pinholes (Figure 12a). The SEM images of the film showed crystal grain size of 80 nm and 200 nm thickness (Figure 12b).

**Figure 13:**
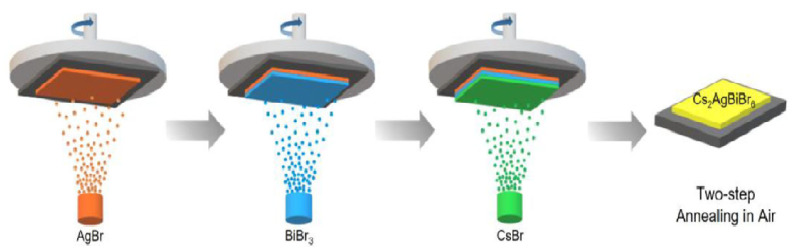
Scheme of sequential vapor deposition processing. Reproduced with permission from [82]. Copyright 2018, Wiley-VCH.

Wang et al. [82] employed the sequential-vapor-deposition procedure for the synthesis of Cs_2_AgBiBr_6_ film as depicted in Figure 13. To obtain an optimized film, annealing was carried out in two different steps. The film was first annealed at 200° for 5 h, which was then followed by annealing at 240° to form the double halide perovskite phase.

**Figure 14:**
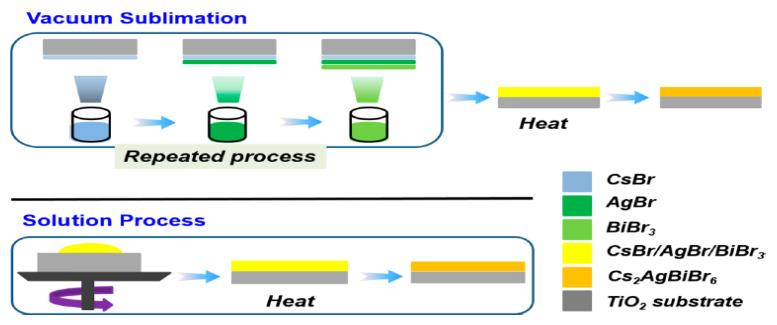
Preparation sketch of Cs_2_AgBiBr_6_ thin film through vacuum-sublimation and solution-processing. Reproduced with permission from [83]. Copyright 2019, American Chemical Society.

In 2019, Igbari et al. [83] selected two different synthesis methods of solution processing and vacuum processing for preparation of Cs_2_AgBiBr_6_ film as shown in Figure 14. The photophysical properties, electronic and crystalline structure as well as photovoltaic properties of the films were compared. The results of various characterizations demonstrated that Cs_2_AgBiBr_6_ film prepared by solution processing possessed higher crystallinity, longer charge carrier lifetime, narrower electronic bandgap of 1.98 eV (2.08 eV for vacuum-processed film) and higher mobility compared with vacuum-processed Cs_2_AgBiBr_6_ film.

#### 2.1.3. Cs_2_M^+^Bi^3+^X_6_: Doping

Doping/alloying process is an effective method for tuning the electronic properties of the semiconducting materials [89]. For perovskite materials the partial of original constituent elements are replaced by targeted ions which leads to diverse advantages including, enhanced optoelectronic performance, stabilized crystal structure, improvement in photoluminescence properties, etc. [90]. A variety of doped Cs_2_M^+^Bi^3+^X_6_ structures are reviewed and listed in Table 3. In 2017, Slavney et al. [91] engineered Cs_2_AgBiBr_6_ double halide perovskite’s bandgap by dilute alloying Tl^+^ (Cs_2_(Ag_1−a_Bi_1−b_)Tl_x_Br_6_; 0.003 ˂ x = a + b ˂ 0.075). DFT-PBE+SOC calculations showed that when Ag^+^ was substituted by Tl^+^ (x = 0.06), the bandgap would decrease by 0.1 eV with direct transition, while replacing the Bi^3+^ with Tl^3+^ (x = 0.06) would lower the bandgap by 0.8 eV and transition would be indirect. Also, doping of 0.075 Tl in (Cs_2_(Ag_1−a_Bi_1−b_) Tl_x_Br_6_ structure decreased the bandgap to 1.40 eV (indirect) or to 1.57 eV (direct). Importantly, the long-lived carrier lifetime in microsecond obtained by time-resolved microwave photoconductivity (TRMC), suggested the efficient extraction of carriers in a solar cell. Both bandgap and carrier lifetime of Cs_2_(Ag_1−a_Bi_1−b_) Tl_x_Br_6_ structure were comparable to MAPbI_3_ values, but the real setback is that as known Tl^+^ is toxic.

**Figure 15:**
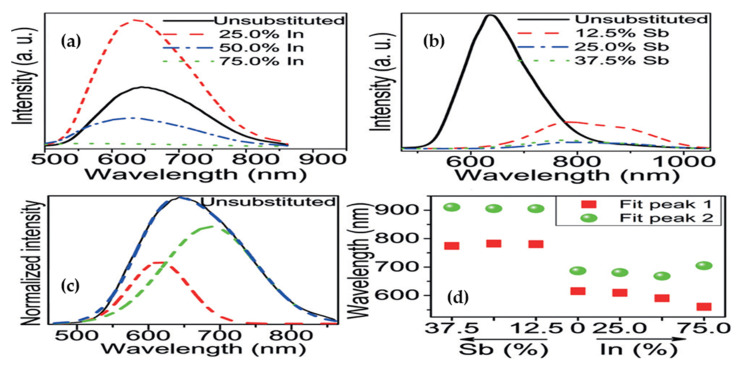
PL spectra for (**a**) In-alloyed; (**b**) Sb-alloyed samples; (**c**) for Cs_2_AgBiBr_6_, fitted using a Gaussian function. Red: fit peak 1, green: fit peak 2, blue dashed line: cumulative fit peak. (**d**) Fitted PL peak positions as a function of Sb and In alloying ratio. Reproduced with permission from [92]. Copyright 2017, Wiley-VCH.

Du et al. [92] through alloying of Sb^3+^/In^3+^ in Cs_2_AgBiBr_6_ perovskite structure, engineered the bandgap and demonstrated long carrier recombination lifetime. Based on UV-vis diffuse spectra, it was shown that by replacing Bi^3+^ with In^3+^, when the level of alloyed metal (x) reach to 0.75 (Cs_2_AgBi_1−x_In_x_Br_6_), the indirect bandgap would increase to 2.27 eV. On the contrary, substitution of Bi^3+^ with Sb^3+^ up to 0.375 (Cs_2_AgBi_1−x_Sb_x_Br_6_; x = 0.375), resulted in narrowing the bandgap from 2.12 eV to 1.86 eV. The opposite bandgap shift direction was related to the different atomic configuration for these metals. The weak PL intensity of In-alloyed sample with high content (x = 0.75) was associated to deeper defect state and symmetry-forbidden transition from valence band to conduction band. Similarly, deep defect state of Sb^3+^-alloyed sample was responsible for the quickly suppressed emission intensity (Figure 15a–d).

Cs_2_AgBi_1−x_In_x_Cl_6_ (x = 0, 0.25, 0.5, 0.75 and 0.9) NCs were prepared by Yang et al. [93], through anti solvent recrystallization. In this work, Cs_2_AgBi_1−x_In_x_Cl_6_ (x = 0.25, 0.5) with indirect bandgap were tuned to direct band gap by increasing the In^3+^ content to 0.75 and 0.9. DFT calculation along with steady-state absorption, PL and transient absorption spectra measurements demonstrated dual color emission of violet (395 nm) (band to band transition) and bright orange (570 nm) (forbidden transition) in NCs.

**Figure 16:**
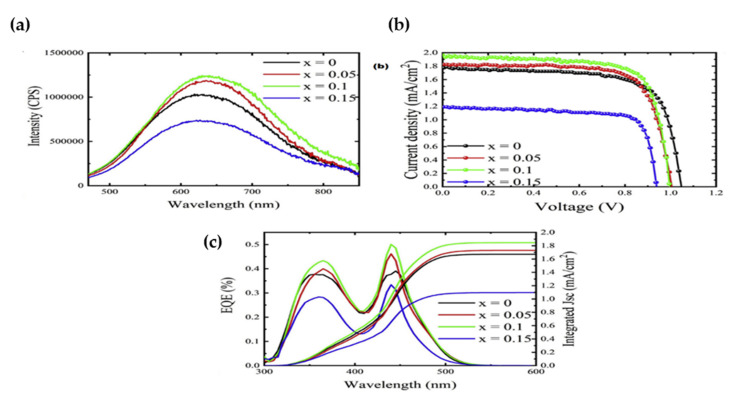
(**a**) The PL spectra of (Cs_1−x_Rb_x_)_2_AgBi_6_ films on quartz; (**b**) The J-V curves of the champion devices with (Cs_1−x_Rb_x_)_2_AgBi_6_ and (**c**) the EQE and IPEC of the devices. Reproduced with permission from [84]. Copyright 2019, Elsevier.

In 2019, Mn^2+^ doped Cs_2_NaBiCl_6_ polycrystalline as a promising orange-red phosphor system was reported by Majher et al. [94]. The absorbed near-UV light by Bi^3+^ ions in the host lattice transferred to Mn^2+^ activators and through the spin-forbidden ^4^T_1 →_^6^A_1_ transition, light emitting from 525 to 700 nm occurred. Also, they [94] demonstrated partial substitution of Cl^−^ ions by Br^−^ resulted in redshift of exciton spectra as well as an optical absorption peak. Zhang et al. [84], through doping of different amount of Rb^+^ ion by replacing Cs^+^ (stochiometric ratio of Cs^+^/Rb^+^: 100/0, 95/5, 90/10, 85/15) prepared different (Cs_1-x_Rb_x_)_2_AgBiBr_6_ perovskite compounds. Optical measurements showed with doping ratio of Cs^+^/Rb^+^: 90:10, the intensity of PL spectra increased due to the reduction of defects. Moreover, based on IPEC spectrum, it was shown that doping of Rb^+^ ion leads in enhancement of absorption at longer wavelength as depicted in Figure 16a–c.

In 2020, Yao et al. [95] reported studies on lead-free double halide perovskite Cs_2_NaBiCl_6_ NCs as host, doped with Ag^+^, Mn^2+^, Eu^3+^ ions through hot-injection approach in order to improve the optical properties of the material. The femtosecond time-resolved transient absorption technique was utilized to investigate the PL enhancement mechanism of ion doped NCs. The excitonic absorption energy of Ag-doped sample exhibited red-shift from 3.82 eV to 3.48 eV which led to significant increase of PLQY from 1.7% to 20%. The Mn^2+^-doped Cs_2_NaBiCl_6_ NCs showed a peak centered in 585 nm along with broad red-orange emission owing to ^4^T_1 →_^6^A_1_ transition of coordinated Mn^2+^ ions. Also, Eu^3+^-doped NCs, owing to the ^5^D_0_ → ^7^F_J_ (J = 1,2,3,4) transition, demonstrated four sharp emission lines at PL spectra corresponding to 591, 615, 652 and 700 nm, respectively.

#### 2.1.4. Cs_2_M^+^Bi^3+^X_6_: Applications

- Photovoltaic Applications

As presented in Table 2, in 2017, Greul et al. [77] fabricated for the first time the Cs_2_AgBiBr_6_-based PSC, which after different parameter measurements exhibited power conversion efficiency (PECs) of 2.43% with V_oc_ exceeding 1 Volt as well as higher stability under constant illumination in ambient environment compared to MAPBI_3_. In another study, Wu et al. [78] fabricated a PSC with Cs_2_AgBiBr_6_ film as active absorbing layer with a PCE of 1.44% and V_oc_ of 1.04 V, J_sc_ of 1.78 mA cm^−2^ and FF of 78 under AM1.5 (100 mW cm^−2^) illumination. The low PCE of the device was attributed to larger exciton binding energy of Cs_2_AgBiBr_6_ than MAPbI_3_. However, the Hole Transporting Layer (HTL)-free device showed higher stability in 4 months compared to MAPbI_3_ based PSCs.

In 2018, Ning et al. [79] demonstrated the Cs_2_AgBiBr_6_ solar cell using one-step spin coating process from single-crystal Cs_2_AgBiBr_6_ solution. The device showed a PCE with maximum value of 1.22% and long photoexcited carrier diffusion length close to 110 nm. The V_oc_ of 1.06 V, J_sc_ of 1.55 mA cm^−2^ and FF of 74 were also achieved for the device. The low PCE value was attributed to the low efficiency of charge extraction by TiO_2_ as the electron transporting layer (ETL) as well as presence of interfacial barrier due to the surface defects. They predicated by increasing the film thickness while preserving the quality of the absorber film and replacing the TiO_2_ by other suitable ETL materials the efficiency of Bi-based halide double perovskite solar cells could be enhanced. Gao et al. [81] adopted anti-solvent dropping methodology for synthesis of Cs_2_AgBiBr_6_ film as already shown in Figure 11a,b. The inverted planer heterojunction fabricated device showed PCE of 2.23% with V_oc_ of 1.01 V, J_sc_ of 3.19 mA cm^−2^ and FF of 69.2 at forward scan as already depicted in Figure 11d,e. The device also showed good reproducibility, negligible hysteresis and long-term stability. Pantaler et al. [80] reported a hysteresis-free solar device using Cs_2_AgBiBr_6_ film as an absorbing layer. In this work, they separately employed three different semiconducting polymers as HTL layer. The best result among different HTL-based devices including spiro-OMeTAD, PCPDTBT (Poly [2,6-(4,4-bis-(2-ethylhexyl)-4H-cyclopenta[2,1-b;3,4-b′]dithiophene)alt4,7(2,1,3-benzothiadiazole)]) and PTAA poly [bis(4-phenyl)(2,4,6-trimethylphenyl)amine), was obtained for PTTA HTL-based device with 1.26% of PCE, 1.02 V of V_oc_ and 1.84 mA cm^−2^ of J_sc_ with FF of 67.

In 2018, Zhang et al. [75] replaced Ag^+^ in Cs_2_AgBiBr_6_ double halide perovskite with Na^+^ ion in order to fabricate the solar device. Cs_2_NaBiI_6_ perovskite was prepared by facile one-step hydrothermal process. However, the fabricated device demonstrated low J_sc_ due to two main reasons, namely that Cs_2_NaBiI_6_ perovskite had (1) low hole transport ability, and (2) was not able to efficiently convert the excitons to current. Nevertheless, the fabricated devices showed high stability for 5 months. Testing of a batch of 20 different devices showed the PCE of 0.42% with V_oc_ of 0.47 V, J_sc_ of 1.99 mA cm^−2^ and FF of 44. Wang et al. [82] fabricated a PSC by employing sequential-vapor-deposition procedure for synthesis of active layer Cs_2_AgBiBr_6_ film as already showed in Figure 13. The device showed lower defect density compered to Cs_2_AgBiBr_6_ film prepared by solution process as well as good stability after 240 h under ambient environment. The PCE of 1.37% and V_oc_ of 1.12 V were reported. By employing solution-based processing and vacuum sublimation method, Igbari et al. [83] could achieve an optimized PCE of 2.51% and 1.41%, respectively for Cs_2_AgBiBr_6_-based PSCs.

In 2020, Wang et al. [64] studied the performance of Zinc chlorophyll (Zn-Chl) as the HTL in Cs_2_AgBiBr_6_-based PSC. It was shown that by employing the Zn-Chl not only enhanced the photovoltaic performance was achieved but also the light absorbing abilities of the Cs_2_AgBiBr_6_ through the sensitizing of the perovskite material was improved. The PSCs based on Zn-Chl showed PCE of 2.79% with V_oc_ of 0.99 V, J_sc_ of 3.83 mA cm^−2^ and FF of 73.6. In another study in 2020, Yang et al. [65] demonstrated that by employing di-tetrabutylammonium *cis*-bis(isothiocyanato) bis (2,2′-bipyridyl-4,4′ dicarboxylato) ruthenium (II) (N719) dye as an interlayer on the surface of Cs_2_AgBiBr_6_ film, the efficiency and stability of Cs_2_AgBiBr_6_-based solar device were boosted. Applying the N719, also led to broadening the light absorption spectrum, reducing the charge carrier recombination, reducing the Cs_2_AgBiBr_6_ film surface defects and accelerating the hole extraction. The optimized solar device showed PCE of 2.84% with V_oc_ of 1.06 V, J_sc_ of 5.13 mA cm^−2^ and FF of 52.4.

In 2021, Wang et al. [66] following their previous work [64], by employing carboxy-chlorophyll derivative (C-Chl) in the mesoporous TiO_2_ film, improved the efficiency of Cs_2_AgBiBr_6_-based solar cells to more than 3%, which is the highest reported efficiency for Cs_2_AgBiBr_6_ PSC. The fabricated PSC based on C-Chl- sensitized mesoporous TiO_2_ film showed improved PCE of 3.11% with V_oc_ of 1.04 V, J_sc_ of 4.09 mA cm^−2^, and FF of 73.

As summarized in this section, Cs_2_AgBiBr_6_-based solar cells have showed low power conversion efficiency compared to lead-based PSCs. However, the results obtained show clearly that Cs_2_AgBiBr_6_ is a promising material for PV application even though the path to enhance the efficiency might be long. The aforementioned studies [77,78,79,82,84] show that employing of a variety of coating engineering such as using Low-pressure assisted method (LPA), anti-solvent method, vapor deposition method and introducing metal ion dopants resulted in enhanced Cs_2_AgBiBr_6_ film morphology improving the efficiency. Furthermore, it was shown that modifying and optimizing ETL and HTL layers also have a significant impact on improvement of efficiency of Cs_2_AgBiBr_6_-based solar cells [64,66].

To increase the fill factor (FF) and the open source voltage (V_oc_), the recombination has to be controlled. It is needed to obtain uniform and high-crystalline perovskite films; meaning that the defect density of the perovskite layer should be reduced [109]. In another words, the higher density traps of the perovskite film cause in more Shockley-Read-Hall (SRH) recombination and lower FF and consequently lead to poor PCE. Wang et al. [66] Used a concept based on ideality factor (*N*) in order to describe the SRH recombination due to defect density. *N* is defined by:

$$N=\frac{e}{K_{B}T}\;\frac{{\mathrm{dV}}_{\mathrm{oc}}}{d\ln I}$$

where *e* is electron charge, *K_B_* is Boltzmann constant, *I* is different light intensity, *T* is the temperature and *N* shows the charge carrier′ recombination process. For ideal solar cells *N* must approach unity. When *N* approaches 2, the performance of the device dominated by Shockley-Read-Hall (SRH) recombination which is assisted by defect density. Therefore, lower *N* value shows suppression of SRH and reduced trap densities which results in higher fill factor and higher PCE [66].

- Non-Photovoltaic Applications

Volonakis et al. [110] adopted first-principle calculation to determine the level of surfaces and surface termination energy of Cs_2_AgBiCl_6_, Cs_2_AgBiBr_6_, Cs_2_AgSbCl_6_ and Cs_2_AgInCl_6_ double halide perovskites. Their investigation demonstrated that according to ionization potential and electron affinity, amongst all these four materials, Cs_2_AgBiCl_6_ and Cs_2_AgBiBr_6_ were the most promising photocatalysts for solar-driven water splitting. Their study also indicated, by increasing the size of halogens in double perovskites, the electron affinity would increase as well, which was due to the shallower energies of the halogen p-states. In 2017, Pan et al. [111] for the first time, reported the application of Cs_2_AgBiBr_6_ single crystals as X-ray detectors. Using of thermal annealing and surface treatment resulted in elimination of disordered Ag^+^/Bi^3+^ and consequently, the resistivity of the crystals improved. The optimized device showed high sensitivity of 105 μC Gy_air_^−1^ cm^−2^, low detection limit of 105 nC Gy_air_^−1^ s^−1^ under the external bias of 5 V as well as long-term operational stability which all are essential for X-ray detectors in order to medical diagnostics. The single crystals of Cs_2_AgBiBr_6_ as suitable semiconductor directly converted X-rays into electrical signals due to its high average atomic number which results in higher X-ray absorption coefficient (α∝Z^4^ /E^3^), adequate μτ product (μ = carrier mobility; τ = carrier lifetime), low ionization energy and high resistivity. Yuan et al. [112] by introducing PEABr (phenylethylamine bromide) into the Cs_2_AgBiBr_6_ perovskite precursors solution, obtained single crystals of Cs_2_AgBiBr_6_ with enhanced ordering degree of [BiX_6_]^3−^ and [AgX_6_]^5−^ in octahedra arrangement. The improved order degree gave rise to lower defect density, tunable bandgap, decreased self-trapped exciton formation and increased carrier mobility. The X-ray detector displayed higher current response of 13 vs. 3190 μs, higher sensitivity of 288.8 μC Gy_air_^−1^ cm^−2^ under a bias of 50 μC Gy_air_^−1^ cm^−2^, higher photoconductive gain and longer carrier drift distance. Li et al. [113] synthesized composites films comprised of Cs_2_AgBiBr_6_ perovskite embedded in a polymer matrix by a simple drop-casting process. Hydroxyl functional groups of polymers significantly increased the dispersity of Cs_2_AgBiBr_6_ in the composite films which led in large area dense films. The fabricated X-ray detector obtained by the composite films demonstrated a sensitivity of 40 μC Gy_air_^−1^ cm^−2^ under the external bias of 400 V, and due to the maximum tolerance of 5% tensile/compressive strain, bending/flexing of detectors did not have any degrading effect on photocurrent.

**Figure 17:**
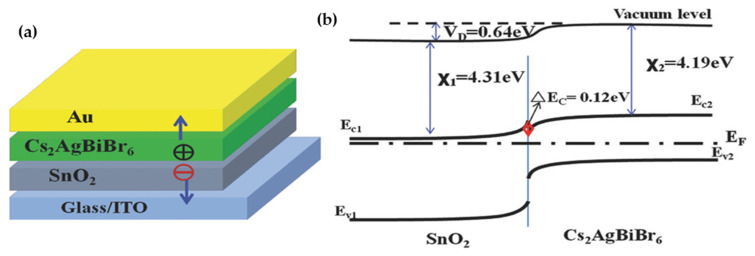
(**a**) Device configuration diagram and; (**b**) Band scheme diagram of Cs_2_AgBiBr_6_/SnO_2_ heterojunction. Reproduced with permission from [115]. Copyright 2018, Wiley-VCH.

Lei et al. [114] adopted a one-step spin-coating synthesis method for preparation of Cs_2_AgBiBr_6_ film as photodetector. The device showed high responsivity of 7.01 A/W, On/Off photocurrent ratio of 2.16 × 10^4^, specific detectivity of 5.66 × 10^11^ Jones, EQE of 2164%, fast response speed of 956/995 μs. The other remarkable feature of unencapsulated photodetector was the high stability under ambient environment against water and oxygen degradation (36 h continuous operation) without no change in photodetection ability. Wu et al. [115] designed a HTL-free planer heterojunction device including ITO/ SnO_2_/ Cs_2_AgBiBr_6_/Au as ultraviolet (UV) photodetector as shown in Figure 17a,b. The self-powered devices exhibited two responsivity peaks at 350 nm and 435 nm which was associated with ultraviolet-A (320–400 nm). The mechanism explained by separation of photogenerated carriers at the of interface of Cs_2_AgBiBr_6_/SnO_2_ heterojunction by its built-in field. A high responsivity of 0.11 A W^−1^ at 350 nm and the quick response of less than 3 ms was comparable with other semiconductor oxide heterojunction-based UV detectors. The unencapsulated UV detector also showed remarkable stability under ambient environment for more than 6 months without any noticeable degradation in photocurrent.

Zhou et al. [116] reported the fabrication of Cs_2_AgBiBr_6_ NCs by hot injection method for CO_2_ photocatalytic reduction. The prepared double halide perovskite demonstrated significant stability against moisture, light and temperature. It also showed a total electron consumption of 105 μmol g^−1^ under simulated solar light (AM 1.5G) for 6 h. Zhang et al. [117] developed an alcohol-based photocatalytic system for dye degradation by applying Cs_2_AgBiBr_6_ double halide perovskite under visible light irradiation. During the photocatalytic process, Cs_2_AgBiBr_6_ kept its high chemical stability in ethanol, and due to the photocatalytic feature of Cs_2_AgBiBr_6_ surface, pseudo-zeroth-order kinetics was obtained, and the reaction rate was also comparable to well-known CdS photocatalyst semiconductor.

### 2.2. Cs/In^3+-^Based Double Halide Perovskites

#### 2.2.1. Cs_2_M^+^In^3+^X_6_: Theoretical Results

**Figure 18:**
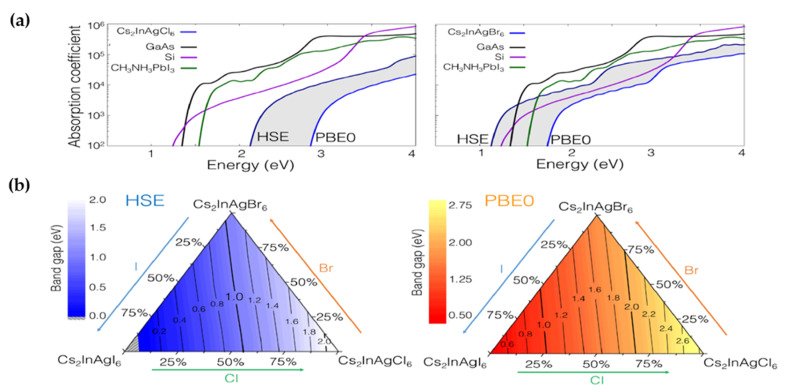
(**a**) Calculated absorption coefficient of the synthesized compound, Cs_2_InAgCl_6_, and hypothetical compound, Cs_2_InAgBr_6_, compared with the theoretical absorption coefficients of Si, GaAs and MAPbI_3_; (**b**) Calculated band gaps of hypothetical mixed-halide double perovskites Cs_2_InAg (Cl_1−x−y_Br_x_I_y_)_6_ within the HSE (left) and PBE0 (right) hybrid functional. The corners of the triangle correspond to Cs_2_InAgX_6_ with X = Cl, Br, I. Reproduced with permission from [118]. Copyright 2017, American Chemical Society.

In 2017, Volonakis et al. [118] by using first-principle calculations, identified the direct bandgap of Cs_2_AgInX_6_ (X = Cl, Br) halide double perovskites. According to DFT/LDA calculation, In-based perovskites showed smaller lattice constant compared to Bi-Based analogous due to the smaller size of In^3+^. Preliminary assessment of octahedral and tolerance factor exhibited that because of smaller ionic radii of In^3+^ (0.8 Å), the coordination between In^3+^ and I^−^ ions would be impossible, and therefore, the synthesis of Cs_2_AgInX_6_ (X = Cl, Br and Cl/Br) double halide perovskite may be amenable. Nominal bandgap for the Cs_2_AgInCl_6_ based on theoretical calculation (HSE and PBE0 hybrid functionals) was reported to be of 2.07 eV with a bias of 0.6 eV (Figure 18a,b). They also demonstrated the VBM was mainly comprised of Cl-3p and In-4d/Ag-4d states while the CBM was occupied by Cl-3p and In-5s/Ag-5s states. The electron and hole effective masses were reported to be 0.20 m_e_ and 0.28 m_h_, respectively.

Zhao et al. [119] as provided in Table 4, inspired by Cu[In,Ga]Se_2_ (CIGS) chalcopyrite solar absorbers [120,121,122], designed 36 different candidates of double halide perovskites where A = Cs, Rb, K; M^+^ = Cu, Ag; M^3+^ = Ga, In and X = Cl, Br, I through first-principles calculations. Although all the compounds showed direct bandgaps, three of them exhibited remarkable bandgap of 1.36 eV (Rb_2_CuInCl_6_), 1.46 eV (Rb_2_AgInBr_6_) and 1.50 eV (Cs_2_AgInBr_6_). Generally, Cu-based compounds showed smaller bandgap than Ag-based perovskites. The screening thermodynamic stability of compounds exhibited positive ∆H_dec_ that led to suppressed decomposition of compounds. Their study [119] showed that by increasing the film thickness up to 2 μm, spectroscopic limited maximum efficiency (SLME) increased to approximately 28%. This value was attributed to small direct bandgaps of Rb_2_CuInCl_6_, Rb_2_AgInBr_6_ and Cs_2_AgInBr_6_ materials which were close to optimal bandgap of 1.34 eV calculated for Shockley–Queisser limit. This SLME value (28%) was comparable to the obtained SLME values for CuInSe_2_ (31.5%) and CH_3_NH_3_PbI_3_ (30%).

The intrinsic defects of In-based double halide perovskite were investigated by Xu et al. [123] through the first-principle calculation. The theoretical study of band structure showed hybridization of Ag(d) and ionic X(p) orbitals along with negligible coupling with In (s) orbital resulted in direct bandgap where both VBM and CBM were placed at Γ point of Cs_2_AgInCl_6_. It was expected that Cs_2_AgInCl_6_ shows more point defects as a quaternary compound, which makes the growth of high-quality film challenging. It was shown that in order to avoid of deep-level defects and unwanted secondary phases, the synthesis of film should be done in Ag-rich growth condition. It was also suggested that depending on chemical growth condition, the conductivity of Cs_2_AgInCl_6_ could change from good n-type/poor n-type to intrinsic semiconducting.

#### 2.2.2. Cs_2_M^+^In^3+^X_6_: Experimental Results

- Cs_2_M^+^In^3+^X_6_: Single-Crystals, Polycrystalline and Nanocrystals-Based Perovskites

In order to verify the accuracy of theoretical studies on stability, direct bandgap and balanced effective masses of Cs_2_AgInX_6_ (X = Cl, Br) Volonakis et al. [118] synthesized the materials through precipitation from the acid solution. The experimental measurements were in a good agreement with their theoretical results. The optical bandgap obtained by experimental measurements was 3.3 eV as presented in Table 4.

Zhou et al. [124] synthesized Cs_2_AgInCl_6_ perovskite crystals by using hydrothermal method. The size of the crystals varied between 5 to 15 μm which was due to the different reaction time. Rietveld analysis for XRD measurement of Cs_2_AgInCl_6_ powder identified the cubic unit cell with the space group of *Fm-3m* [AgCl_6_] and [InCl_3_] octahedra in 3D framework. Time-resolved emission spectra showed two different decay times of 16.3 and 100 μs which was attributed to surface/defect states and fundamental nonradiative recombination. The optical bandgap measured by UV-vis reflectance spectroscopy was 3.23 eV while this value determined to be 3.33 eV by using band structure and optical absorption calculations. However, the compound showed to maintain high light, moisture and heat stability.

#### 2.2.3. Cs_2_M^+^In^3+^X_6_: Doping

In 2018, Nag et al. [96] prepared bulk Mn-doped Cs_2_AgInCl_6_ perovskite employing the same method used by Volonakis et al. [118] in order to study the photoluminescence properties. The optical measurements of samples showed a weak intensity of undoped sample while by increasing of Mn^2+^ concentration as dopant, the intensity of emissions raised up significantly. This was due to the de-excitation of Mn^2+^ d-electrons from ^4^T_1 →_^6^A_1_ state. Locandi and co-workers [97] prepared Cs_2_AgInCl_6_ and Mn-doped Cs_2_AgInCl_6_ NCs, using colloidal hot-injection method. Synthesis resulted in highly pure NCs without any undesired secondary phases for both Cs_2_AgInCl_6_ and Mn-doped Cs_2_AgInCl_6_ as well as high thermal stability up to 500°. The experimentally obtained optical bandgap was larger compared to previously reported works (4.38 eV for Cs_2_AgInCl_6_ NCs and 4.36 eV for Mn-doped Cs_2_AgInCl_6_ NCs). However, the Mn-doped Cs_2_AgInCl_6_ NCs exhibited bright PL emissions with a PLQY of 16 ± 4% which is comparable to Cs_2_AgInCl_6_ NCs value (1.6 ± 1%). This result shows that doping with Mn^2+^ would make the In^3+^-based double halide perovskites a good candidate for different applications such as LEDs.

**Figure 19:**
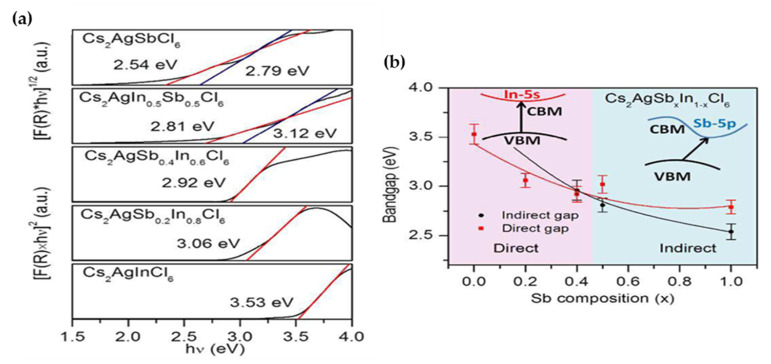
(**a**) Tauc plots for Cs_2_AgSbCl_6_, Cs_2_AgSb_0_._5_In_0_._5_Cl_6_, Cs_2_AgSb_0.4_In_0.6_Cl_6_, Cs_2_AgSb_0.2_In_0.8_Cl_6_ and Cs_2_AgInCl_6_. The plots indicate the characteristics of an indirect bandgap for Cs_2_AgSbCl_6_, a direct bandgap for Cs_2_AgInCl_6_ and a transition from indirect to direct bandgap of Cs_2_AgSb_x_In_1__−x_Cl_6_ at x = 0.4; (**b**) Phase diagram of Cs_2_AgSb_x_Ag_1__−x_Cl_6_ showing the bandgap trend as a function of Sb composition and a crossover from indirect to direct optical absorption in a solid solution at x = 0.4. Reproduced with permission from [125]. Copyright 2017, Royal Society of Chemistry.

Tran et al. [125] through a solid-state technique synthesized Cs_2_AgInCl_6_ while the Cs_2_AgSb_x_In_1−x_Cl_6_ (x = 0.5, 0.4 and 0.2) solid solutions were prepared by combining the single crystals of hydrothermally synthesized of Cs_2_AgInCl_6_ and Cs_2_AgSbCl_6_, in a stoichiometric ratio. UV-Vis diffuse reflectance measurement along with Tauc plot demonstrated, by increasing the Sb composition in Cs_2_AgSb_x_In_1−x_Cl_6_ (x = 0.5, 0.4 and 0.2 and 0), the bandgap would shift from direct to indirect while the value decreased from 3.53 eV to 2.54 eV in Cs_2_AgSbCl_6_ (Figure 19a,b).

Liu et al. [99] by utilizing a facile hot injection method, prepared Cs_2_AgInCl_6_ and Bi-doped Cs_2_AgInCl_6_ NCs. It was indicated that by controlling the reaction time both ligands and HCl concentration, the purer NCs without any impurity phases with desirable size and shapes would be produced. By increasing the reaction temperature from 180° to 280°, the PLQY curve also rise and reached to highest PLQY of 11.4%. TEM and HRTEM analysis of NCs formed at 180° exhibited of some spherical particles which were ascribed to Ag_2_O. Due to metal-ion-induced oxidation process the silver ions turn to silver nanoparticles. The size of undoped and doped (with 1% Bi^3+^ ions) NCs were reported 9.79 nm and 10.59 nm, respectively. The optical measurements of NCs revealed a broad orange peak at 580 nm for Bi-doped Cs_2_AgInCl_6_ and a blue emission peak at 470 nm for Cs_2_AgInCl_6_. It was also indicated that by doping Bi^3+^ in Cs_2_AgInCl_6_ NCs, the number of defects would decrease, and radiative localization would be promoted. The long-life time and broadened emission of Bi-doped Cs_2_AgInCl_6_ NCs were attributed to self-trapped excitation (STEs) stemming from the Jahn-Teller distortion of [AgCl_6_] octahedron in the excited state as well as the trivial sub-bandgap defect state transition.

In 2019, Hu et al. [98] prepared colloidal Cs_2_Ag_1−x_Na_x_In_1−y_Bi_y_Cl_6_ (x = 0–1; y = 0.03–0.16) NCs by a room temperature recrystallization process. The mean size of NCs were reported to be of 3 nm with diameter of 1.8–4.0 nm which could potentially confine the excitons. The remarkable blueshift photoluminescence was observed for the NCs which was comparable for bulk materials. Incorporation of partial amount of Na^+^ and Bi^3+^ resulted in bright near-white light emission with tunable color temperatures from 9759.7 to 4429.2 K. Furthermore, the introduction of Bi^3+^ ions along with OA (ligand) passivation in Cs_2_Ag_0.17_Na_0.83_In_0.88_Bi_0.12_Cl_6_ nanocrystals led in PLQY of 64% which was the highest reported value for lead-free NCs to date.

In order to investigate the geometric, electric and photoluminescence properties of Mn^2+^-doped Cs_2_AgInCl_6_ double halide perovskite, Wu et al. [126] employed first-principle calculations. Their study showed that the presence of Mn as a dopant resulted in defect complexes by replacement of Ag^+^ with Mn^2+^ atom and causing Ag vacancy (Mn_Ag_ V_Ag_), which was due to the charge balance and weak distortion of the metal octahedra. Subsequently, this defect configuration introduced two defect bands in the forbidden gap which was associated to 3d orbitals of the Mn^2+^ ions. Therefore, the transition of electron from the first excited state to the ground state led to lower PL spectrum compared to bandgap which would make it beneficial for LEDs.

In 2019, in order to tune the bandgap of Cs_2_AgInCl_6_, microcrystal and colloidal nanocrystals of Yb^3+^ doped Cs_2_AgInCl_6_ were prepared by Mahor et al. [100] through the solution-process and hot-injection methods, respectively. The concentration of Yb^3+^ content, analyzed by Inductively coupled plasma mass spectrometry (ICP-MS) was reported to have 0.1–1.6% for microcrystals and 6.2% for NCs. The Yb-doped samples showed an intense NIR emission centered at ~994 nm. Optical measurements of the samples indicated the light at first was absorbed by the light and then non-radiatively transferred to excite the Yb^3+^ ions and resulted in the de-excitation of (f) electrons in Yb^3+^ ions (^2^F_5/2_ → ^2^F_7/2_). However, the PL decay was different for microcrystals compared to nanocrystals doped perovskites. The NCs exhibited a biexponential decay of 3 ms and 749 μs, and the microcrystals showed a single-exponential decay of Yb-emission with a lifetime of 2.7 ms. The samples also showed high stability under ambient environment.

In 2020, the Pb-free double halide perovskite Cs_2_NaInCl_6_:Sb^3+^ was prepared by Gray et al. [101] through precipitation from an HCl solution with the aim of photoluminescent properties investigation. The PXRD analysis of doped and undoped compounds indicated *Fm-3m* crystal symmetry, *a* = 10.553344(4) Å with rock salt fully ordering of In^3+^ and Na^3+^ ions sites. The compound showed a large bandgap of ~5.1 eV. However, the substitution of In^3+^ with Sb^3+^ resulted in strong absorption in the UV because of 5s^2^ → 5s^1^5p^1^ transitions of [SbCl_6_]^3−^. Through the transition from ^3^P_1_ → ^1^S_0_ with radiative relaxation back to 5s^2^ ground state, strong blue luminescence at 445 nm with a PLQY of 79% was observed. Nevertheless, with increasing of Sb^3+^ content in Cs_2_NaInCl_6_, more than 3% the PL intensity decreased. Furthermore, Cs_2_NaInCl_6_:Sb^3+^ showed smaller Stocks shift (0.94 and 1.38 eV) compared to vacancy ordered double perovskite Cs_2_SnCl_6_, which was due to the change of coordination number from 6 in Cs_2_NaInCl_6_ to 5 in Cs_2_SnCl_6_.

In a similar study, Zeng et al. [102] showed that doping of 10% Sb^3+^ in Cs_2_NaInCl_6_ perovskite would break the parity forbidden transition as well as modulating of density of state (DOS) population which led to an optimized blue PLQY of 78.9% assigned to be self-trapped excitons (STEs).

#### 2.2.4. Cs_2_M^+^In^3+^X_6_: Applications

- Non-Photovoltaic Applications

In 2017, Luo et al. [127] fabricated single crystals of Cs_2_AgInCl_6_ by thermodynamic synthesis method for UV photodetection. The prepared single crystals showed light yellow color on surface with colorless interior. This phenomenon was explained by different compositions of sample which means oxygen or oxygen containing functional groups change the surface compositions, consequently the optical properties of sample would change. Furthermore, the optical measurements of sample verified the existence of parity-forbidden transition in Cs_2_AgInCl_6_, which had been already demonstrated by Yan et al. [53] through theoretical calculation. It was suggested that the large difference between experimentally obtained optical bandgap (3.2 eV) and photoluminescence emission energy (2.1 eV) was caused by parity-forbidden transitions. However, the fabricated UV photodetection device exhibited ultralow trap-density of 8.6 ± 1.9 × 10^8^ cm^−3^ which was comparable with Pb-based perovskites value (1.80 ± 1.07 × 10^9^ cm^−3^), as well as high ON-OFF ratio of around 500, fast photo response of 1 ms, low dark current of 10 pA at 5 V bias and high detectivity of 10^12^ Jones.

Co-doped Cs_2_AgInCl_6_: Bi^3+^-Ln^3+^ (Ln^3+^ = Er^3+^ and Yb^3+^) perovskite, with the aim of improving both absorption and emission spectra of the Cs_2_AgInCl_6_ were prepared by Arfin et al. [128]. Their study showed that co-doping of Bi^3+^ in Cs_2_AgInCl_6_ along with Ln^3+^(Ln^3+^ = Er^3+^ and Yb^3+^) resulted in new optical absorption channel in a lower energy (372 nm), which is appropriate for excitation in commercial UV LEDs. This excited energy gets effectively transferred to f-electrons of Er^3+^ or Yb^3+^ ions leading to emission at 1540 nm and 994 nm, respectively and generally improve the near infrared (NIR) dopant emissions. Moreover, excitation at lower energy led to that co-doped Bi^3+^-Er^3+^: Cs_2_AgInCl_6_ exhibited 45-times higher emission intensity and co-doped Bi^3+^-Yb^3+^: Cs_2_AgInCl_6_ showed 27-times higher emission intensity compared to single doped Er^3+^: Cs_2_AgInCl_6_ and Yb^3+^: Cs_2_AgInCl_6_ compounds.

### 2.3. Cs/Sb^3+^-Based Double Halide Perovskite

#### 2.3.1. Cs_2_M^+^Sb^3+^X_6_: Theoretical Results

In 2017, Tran et al. [125], as presented in Table 5, exhibited a new design for engineering the convergence of direct and indirect bandgap in double halide perovskites based on chemical adjustment of (s) and (p) orbitals character in CBM. Because of differences in orbital overlaps, the relative crystals momenta of VBM and CBM determine whether a bandgap is direct or indirect. This means that bands derived by s-orbitals will increase in energy from Γ to X in a cubic Brillion zone while the bands derived by p-orbitals reduce in energy. Therefore, if the conduction band can be adjusted from s orbitals to p orbitals with a negligible change in the valance band consequently, the difference would result in shift from direct to indirect bandgap. By means of this theory, they examined the feasibility of their design strategy with the experimental preparation of Cs_2_AgInCl_6_, Cs_2_AgSbCl_6_ and Cs_2_Sb_x_In_1−x_Cl_6_ (x = 0.5, 0.4 and 0.2). Their experimental study successfully demonstrated that Cs_2_AgInCl_6_ and Cs_2_AgSbCl_6_ showed direct and indirect bandgaps, respectively. By employing the optimized amount of 60%: 40% for In^3+^: Sb^3+^ in Cs_2_Sb_x_In_1−x_Cl_6_, it was shown that the bandgap changes from indirect to direct.

Zhou et al. [130] investigated the color-tuning phenomenon of Cs_2_AgSbCl_6_ crystals which occurred during the synthesis procedure by PBE approaches through first-principle calculation while anti-site defect was established as a model. It was shown that with exchanging site-equal Ag and Sb ions, the two allotropes of nearest neighbor (NN) and second nearest neighbor (2NN) were stable with only 7–12 meV per atom larger than balanced structure (E_balanced_ > E_NN_ > E_2NN_), and the relatively small lattice expansion resulted in different bandgaps.

In 2019, Wei et al. [62] investigated the crystallinity and symmetry of Cs_2_AgSbBr_6_ double halide perovskites by employing DFT calculations. It was shown that even though there was a similarity between band structure of Cs_2_AgSbBr_6_ and Cs_2_AgBiBr_6_, the 5s 5p orbitals in Sb lowered the CBM significantly, which led to smaller bandgap in Cs_2_AgSbBr_6_ compared to Cs_2_AgBiBr_6_. Lin et al. [68] presented a strategy for developing quadruple perovskite halides. Through DFT calculations and symmetry analysis, Cs_4_CdSb_2_Cl_12_ and Cs_4_CdBi_2_Cl_12_ were identified as two stable perovskites with vacancy ordered 3D crystal structure along with 3D electronic dimensionality with direct forbidden bandgaps.

#### 2.3.2. Cs_2_M^+^Sb^3+^X_6_: Experimental Results

- Cs_2_M^+^Sb^3+^X_6_: Single-Crystals, Polycrystalline and Nanocrystals-Based Perovskites

**Figure 20:**
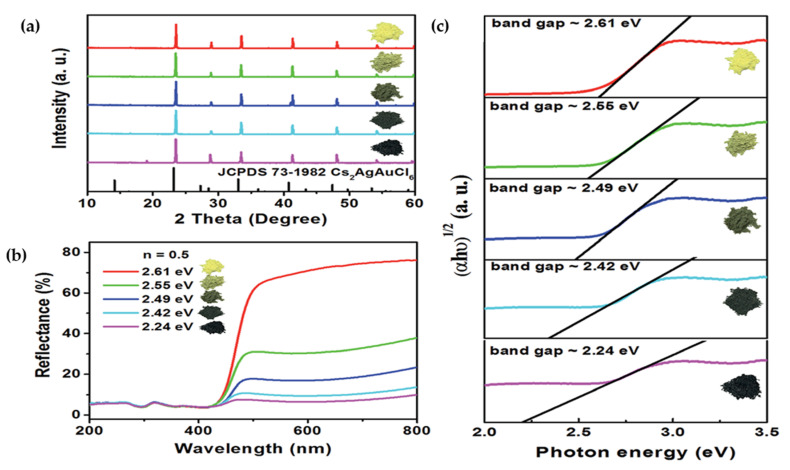
(**a**) XRD pattern; (**b**) UV-vis diffuse spectra and; (**c**) Tauc plots of as-synthesized Cs_2_AgSbCl_6_ samples obtained by employing of different amounts of HCl (0.5, 0.75, 1.0, 1.25 and 1.5 mL) with variable visible light absorbance. Reproduced with permission from [130]. Copyright 2018, Royal Society of Chemistry.

Zhou et al. [130] hydrothermally synthesized Cs_2_AgSbCl_6_ crystals and demonstrated by increasing the amount of HCl as a solvent from 0.5 to 1.5 mL, the color of prepared powders changed from yellow to near black, which directly influenced on bandgap. While the darker samples showed lower bandgaps and the lighter color samples indicated higher bandgap as depicted in Figure 20a–c.

Vargas et al. [129] by incorporation of Cu^2+^ and Sb^3+^ through a solution method, prepared a 2D layered double halide perovskite, Cs_4_Sb_2_CuCl_12_, which exhibited a promising direct bandgap of around ~1 eV, due to the unpaired electron in the 3d orbital of Cu^2+^. The crystalline structure of the perovskite comprised of [CuCl6]^4−^ octahedra which was placed between [SbCl6]^3−^ layers and corner-shared to [SbCl6]^3−^ octahedra. The prepared perovskite showed high stability against moisture, light and temperature.

In 2019, Wei et al. [62] prepared a bulk form of Cs_2_AgSbBr_6_ double halide perovskite through the hydrothermal method. The prepared samples showed a low indirect bandgap of 1.64 eV for single crystals. The color of as-prepared samples changed from black to brown by increasing the temperature to 480 K for 5 min, which could be ascribed to charge transfer between Sb^3+^ and Sb^5+^ in a black phase.

**Figure 21:**
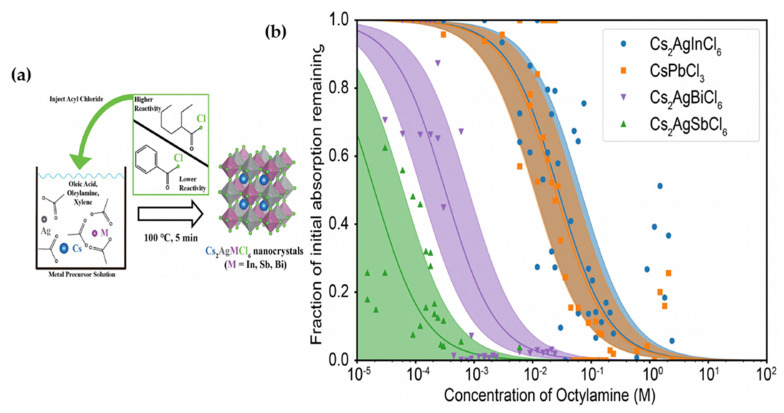
(**a**) reaction sketch for the preparation of double halide Cs_2_AgSbCl_6_, Cs_2_AgInCl_6_ and Cs_2_AgBiCl_6_ perovskite NCs. A solution of metal acetates (cesium, silver and indium, antimony or bismuth) in xylene with oleic acid and oleylamine is heated to 100 °C, then an acyl chloride precursor is injected to form NCs of the corresponding Cs_2_AgMCl_6_ (M = In, Sb, Bi) double perovskite. The reactivity of the acyl chloride precursor is an important factor for tuning nanocrystal formation; (**b**) analysis of stability for Cs_2_AgSbCl_6_, Cs_2_AgInCl_6_ and Cs_2_AgBiCl_6_ and CsPbCl_3_ NCs. Individual points represent the fraction of initial absorption remaining after 4 h as a function of the different concentrations of octylamine that the nanocrystal solutions were exposed to. Reproduced with permission from [85]. Copyright 2019, American Chemical Society.

Dahl et al. [85] synthesized Cs_2_AgSbCl_6_ and Cs_2_AgInCl_6_ nanocrystals by using a modified hot injection method. Instead of adding cesium oleate which is regularly used in the synthesis of double halide perovskites acyl halides was added into the solution of metal acetate precursors under ambient environment at mild temperatures. It was found that the concentration and type of acyl halide had a deep effect on synthesized nanocrystals. The prepared crystalline nano-cubes showed an edge length of 10 nm terminated with (200) facets as well as small silver nano-crystallites decorating the cubes. By developing a titration essay, in order to test the stability of prepared NCs, Dahl et al. [85] observed that Cs_2_AgSbCl_6_ dissolved in the presence of minimum concentration (0.01–0.1 mM) of octylamine and released more than twice decomposition energy compared to Cs_2_AgInCl_6_ and CsPbCl_3_ as illustrated in Figure 21a,b.

Lin et al. [68] synthesized Cs_4_CdSb_2_Cl_12_ and Cs_4_CdBi_2_Cl_12_ 3D perovskites by solvothermal method in order to support their theoretical results. The steady-state PL exhibited warm orange emission, while the transient PL showed carrier recombination lifetime of microseconds at low temperature.

Garcia-Espejo et al. [63] by employing a mechanochemical approach, prepared polycrystalline inorganic Cs_2_AgSbBr_6_ double perovskites. Bromide derivative salts were added to a high energy ball mill with different molar ratio under atmospheric condition. Due to the thermodynamic instability of Cs_2_AgSbBr_6_, XRD for Cs_2_AgSbBr_6_ showed varied X-ray diffraction peaks showing the formation of side phases such as AgBr, CsAgBr_2_ and Cs_3_Sb_2_Br_9_. It was demonstrated that 2D layered of Cs_3_Sb_2_Br_9_ is more stable than 3D Cs_2_AgSbBr_6_ double perovskite [131]. The Cs_2_AgSbBr_6_ bandgap from Tauc plot was estimated to be 1.93 eV.

Deng et al. [132] integrated Cs_2_AgSbCl_6_ powder and Cs_2_AgSbCl_6_/TiO_2_ heterojunction nanoparticles through solution state method and hydrothermal process, respectively. Optical bandgaps of 2.60 eV from optical absorption curve for Cs_2_AgSbCl_6_ were obtained. However, the optical absorption was seen improved for Cs_2_AgSbCl_6_/TiO_2_ heterojunction sample in the visible region as a result of interface states formation and lowered bandgap showing the facilitation of the photo-induced optical transitions. The comparison of charge transfers of Ag_2_Sb_2_Cl_8_/TiO_2_ and Cs_4_Cl_4_/TiO_2_ indicated that photo-induced carrier separation was more efficient at Cs_4_Cl_4_/TiO_2_ interface.

#### 2.3.3. Cs_2_M^+^Sb^3+^X_6_: Doping

Tran et al. [125] through the solid-state technique synthesized Cs_2_AgSbCl_6_ perovskite and Cs_2_AgSb_x_In_1__−x_Cl_6_ (x = 0.5, 0.4 and 0.2) were prepared by combining the single crystals of hydrothermally synthesized Cs_2_AgInCl_6_ and Cs_2_AgSbCl_6_ in a stoichiometric ratio. UV-Vis diffuse reflectance measurement along with Tauc plot demonstrated that by increasing the Sb composition in Cs_2_AgSb_x_In_1__−x_Cl_6_ (x = 0.5, 0.4 and 0.2 and 0), the bandgap would shift from direct to indirect while the value decreased from 3.53 eV to 2.54 eV in Cs_2_AgSbCl_6_.

**Figure 22:**
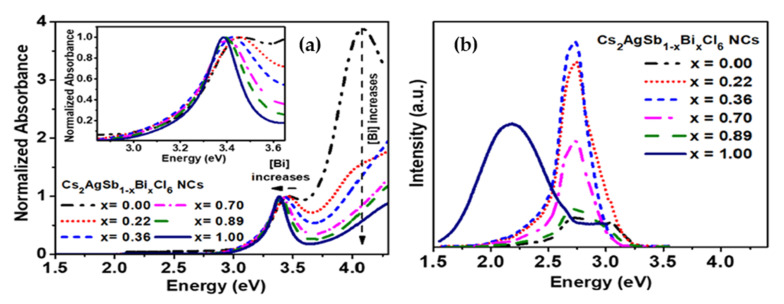
(**a**) UV-vis absorption and; (**b**) PL spectra after excitation at 310 nm for colloidal Cs_2_AgSb_1−x_BixCl_6_ alloy NCs. The samples in (**a**) all were normalized at 3.4 eV which is the lowest energy peak. Reproduced with permission from [104]. Copyright 2019, American Institute of Physics.

Karmakar et al. [103] by investigating of Cs_2_AgSbCl_6_ and Cu^2+^-doped Cs_2_AgSbCl_6_ double halide perovskites reported of a well- ordered crystal structure with integration of Cu^2+^ ions into the lattice where Ag^+^ ions were replaced by Cu^2+^. The results of optical measurements showed that Cu^2+^ ions had a direct effect on reduction of bandgap from 2.56 eV (Cs_2_AgSbCl_6_) to 1.02 eV for Cu-doped Cs_2_AgSbCl_6_ (x = 0.1 Cu^2+^). The prepared Cu-doped Cs_2_AgSbCl_6_ perovskite exhibited significant stability up to 365 days. The DFT calculation demonstrated small carrier effective masses (>0.4 m_e_). Furthermore, it was found that using Cu^2+^ ion as dopant increased the conductivity of semiconductor. Kshirsagar et al. [104] prepared Cs_2_AgSb_1__−x_Bi_x_Cl_6_ alloy NCs with 0 ≤ x ≤ 1 by employing the hot-injection method. Incorporation of Bi^3^ in Cs_2_AgSb_1-x_Bi_x_Cl_6_ NCs (x = 0.36) raised the PL emission intensity to the maximum level of 2.74 eV. Also, a broadened red-shift emission was observed at 2.17 eV which was ascribed to the carrier-phonon coupling leading intrinsic self-traps. The average length of 10 nm was characterized by TEM images, which proved that even with incorporation of Bi, the size and shape of NCs remained unchanged. The absorption spectra of Cs_2_AgSbCl_6_ exhibited an absorption peak at 3.45 and 4.08 eV. Since the edge length of synthesized NCs were larger than Bohr radius (1.02 nm), and due to the absence of quantum confinement effect in NCs, no modifications in absorption spectrum were observed. However, the addition of Bi in Cs_2_AgSb_1__−x_Bi_x_Cl_6_ (x = 1), decreased the bandgap from 3.45 eV to 3.39 eV as a result of larger spin-orbit coupling strength of Bi as well as anti-site disorders as shown in Figure 22a,b.

#### 2.3.4. Cs_2_M^+^Sb^3+^X_6_: Applications

- Photovoltaic Applications

To the best of our knowledge, there have not been any significant studies on Sb^3+^-based double perovskite for PV application. The only fabricated PSC based on Cs_2_AgSbBr_6_ thin film was reported by Wei et al. [62] in 2019 with a very low photovoltaic efficiency (0.01%), which was attributed to the presence of secondary phases with large bandgaps.

### 2.4. Cs-Based Vacancy-Ordered Double Halide Perovskites

**Figure 23:**
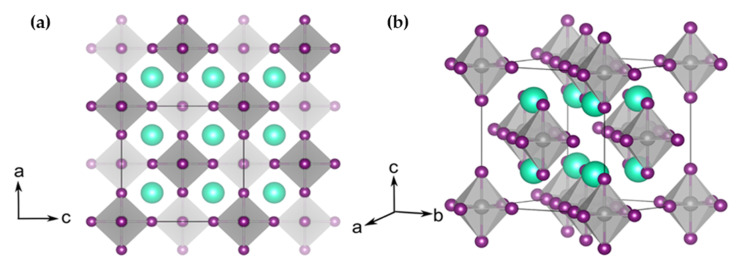
(**a**) Crystal structure of the vacancy-ordered double Cs_2_SnI_6_ and Cs_2_TeI_6_ perovskites; (**b**) Reorientation of the unit cell reveals the isolated octahedral units. Reproduced with permission from [136]. Copyright 2016, American Chemical Society.

Vacancy-ordered double perovskites are another form of double perovskite in which one B-site cation is replaced by a vacancy and the other B-site cation is in a oxidation state of B^4+^ (A_2_M^1+^M^3+^X_6_ → A_2_□M^4+^X_6_ → A_2_M^4+^X_6_; where □ indicates a vacancy). This category of perovskite materials also has the close-packed anionic lattice like ABX_3_. While in ABX_3_ structure, the stability of the perovskite is predicted by Goldschmidt tolerance factor, in A_2_BX_6_ structures the radius ratio is calculated by A-site cation radius to the 12-coordinate void [133,134,135,136] as shown in Figure 23a,b.

#### 2.4.1. Cs_2_M^4+^X_6_: Theoretical Results

In 2014, Lee et al. [137], as provided in Table 6, investigated the band structure of perovskite by DFT calculations and it was shown that Cs_2_SnI_6_ possessed a direct bandgap of 1.3 eV at the Γ point comprised of filled I-5p and empty I-6p/Sn-5p orbitals which contributed to VBM and CBM, respectively. Both VBM and CBM were dispersed in energy resulting in high charge carrier mobility of Cs_2_SnI_6_.

Maughan et al. [106] through DFT calculations indicated that the phonon interaction was stronger in Rb_2_SnI_6_ compared to Cs_2_SnI_6_. This was caused by the larger number of nondegenerate lower frequency phonons which contributed to the lattice dielectric response and decreased charge carrier mobilities. Debbichi et al. [138] theoretically studied the atomic and electronic band structures of single crystal and polycrystal Cs_2_Au_2_I_6_. It was shown that Au demonstrated mixed-valence of +1 and +3 in the B-site, which made the Cs_2_Au_2_I_6_ to exhibit the same electronic structure as double perovskite materials resulting in an optimal bandgap of 1.31 eV. An in-depth optical simulation done by Debbichi et al. [138] suggested that polycrystalline Cs_2_Au_2_I_6_ would be a good candidate for PV application because of its remarkable optical absorption and electrical performance such as small effective masses and long diffusion length. It was predicted by employing polycrystalline Cs_2_Au_2_I_6_ as an active layer in PSC, the short-circuit-current of 30 mA cm^−2^ and photoconversion efficiency of 20% could be obtained. They used full-wave electromagnetic simulations based on the finite element methods (FEM) to do their calculation.

In 2017, Ju et al. [139] introduced a new class of vacancy-ordered perovskite based on Ti^4+^ ion. Their experimental and theoretical results indicated that both Cs_2_TiI_2_Br_4_ and Cs_2_TiBr_6_ possessed the ideal bandgap values of 1.38 eV for single junction and 1.78 eV for tandem solar cells. Moreover, both materials exhibited good environmental stability. Sakai et al. [140], by employing DFT/GCA calculations showed that the electron and hole effective masses of Cs_2_PdBr_6_ were to be 0.53 and 0.85 m_e_, respectively, which resulted in n-type semiconductivity in Cs_2_PdBr_6_.

#### 2.4.2. Cs_2_M^4+^X_6_: Experimental Results

- Cs_2_M^4+^X_6_: Single-Crystals, Polycrystalline and Nanocrystals-Based Perovskites

Lee et al. [137] prepared Cs_2_SnI_6_ perovskite as a stable molecular iodosalt serving as a hole transporting layer in solid-state DSSCs. This iodosalt compound which Sn is on its +4-oxidation state showed high stability against moisture and air compared with CsSnI_3_ and MASnI_3_ perovskites.

In 2019, Kong et al. [141] adopted a solution-based process for synthesis of Cs_2_TiX_6_ (x = Cl and Br) at room temperature. The highly uniform and thermally stable crystals and thin film of Cs_2_TiBr_x_Cl_1−x_ (0 < x < 1) were prepared. The obtained materials showed a quasi-direct bandgap of 1.7 eV for Cs_2_TiBr_6_, 1.95 eV for Cs_2_TiBr_2_Cl_4_ and 2.5 eV for Cs_2_TiCl_6_. Furthermore, steady-state PL exhibited an emission peak centered at ~535 nm for Cs_2_TiCl_6_, ~635 nm for Cs_2_TiBr_2_Cl_4_ and ~670 nm for Cs_2_TiBr_6_. The FWHM values for all prepared samples were larger than 100 nm, which is comparable with Pb-based perovskites. However, in order to obtain more efficient thin films and for engineering the bandgaps, they suggested different synthesis approaches and alloying with other metallics or halide elements were required. Sakai et al. [140] by employing the solution process technique, synthesized Cs_2_PdBr_6_ halide perovskite. During the synthesis process, the oxidation state of Pd^2+^ changed to Pd^4+^ which was generated in situ. The obtained compound crystallized in a cubic structure with a space group of *fm-3m*. The optical measurements indicated that compound had an indirect bandgap of 1.6 eV. In order to study the sample photoconductivity a sandwich structure was fabricated including ITO/Cs_2_PdBr_6_/Ag. The carried-out experiment confirmed the feasibility of Cs_2_PdBr_6_ perovskite for different optoelectronic applications such as LEDs, PV and photon-sensors.

- Cs_2_M^4+^X_6_: Film-Based

Chen et al. [87], as already presented in Table 2, by employing the facile low-temperature vapor deposition methodology, prepared a high-quality thin film of Cs_2_TiBr_6_ halide perovskite. The synthesized thin film demonstrated an optimal bandgap of 1.78 eV with a carrier diffusion length of more than 100 nm.

#### 2.4.3. Cs_2_M^4+^X_6_: Doping

In 2016, the solid-solution of Cs_2_Sn_1−x_Te_x_I_6_ was prepared by Maughan et al. [136] in order to investigate of its structure-property relationship. The substitution of Sn by Te causing the increase of electronic dispersion due to the closer contact distances of I-I bonding resulted in reduction of conductivity, carrier mobility and carrier concentration. DFT calculation revealed the hindered formation of intrinsic iodine vacancy donor defects resulting in insulating characteristics of Cs_2_TeI_6_. However, the theoretical calculation of Cs_2_SnI_6_ native defects showed a low enthalpy of iodine vacancies formation and a level of defect energy that was a shallow donor to the conduction band. This makes the material tolerant to defect states. In Te-doped compound, the covalency of Te-I bonding suppresses the formation of iodine vacancy state causing the reduction in conductivity.

Maughan et al. [106] studied the electrical and structural changes created by substitution of Cs^+^ with Rb^+^. Both Cs_2_SnI_6_ and Rb_2_SnI_6_ showed a native n-type semiconductivity. However, the replacement of Cs^+^ with Rb^+^ decreased the charge carrier mobility by ~50 times compared to Cs_2_SnI_6_ that makes Cs_2_SnI_6_ as an interesting material for optoelectronic applications.

In 2018, Tan et al. [107] selected vacancy-ordered Cs_2_SnCl_6_ perovskite as a host and Bi^3+^ as the luminescence dopant. The prepared Cs_2_SnCl_6_: Bi perovskite showed an intense rise of PLQY (78.9%) with an emission peak at 445 nm. Furthermore, the doping of Bi resulted in narrowing of bandgap from 3.9 eV in Cs_2_SnCl_6_ to 3.0 eV in Cs_2_SnCl_6_: Bi due to the generating of defect bands [Bi_Sn_ + V_Cl_]. The formation of BiOCl layer as well as stable oxidation state of Sn^4+^ enhanced the thermal and water stability. Also, the combination of Cs_2_SnCl_6_: Bi with commercial yellow phosphors along with commercial UV-LED chips led to high warm light emission with a correlated color temperature of 4486 K and a commission Internationale de I’Eclairage (CIE) coordinate of (036; 0.37).

In 2019, Ma et al. [108] studied the effect of Ge^4+^ substitution on CsSn_1__−x_Ge_x_I_6_ (x = 0.25, 0.5, 0.75, 1) properties. First-principle calculation demonstrated that the concentration of Ge^4+^ and the value of the bandgap had linear relationship. The crystal cells of traditional ABX_3_ perovskites were easily influenced by doping small cations which caused tilting and contraction of the cell [142]. In this study, doping of Ge did not result in tilting up the BI_6_ octahedra, but the contraction of crystal cell occurred, which gave rise to the reduction of bandgap. Employing DOS and PDOS calculations, it was shown that doping of Ge^4+^ led to the change in composition of CBM and the bandgap.

#### 2.4.4. Cs_2_M^4+^X_6_: Applications

- Photovoltaic applications

**Figure 24:**
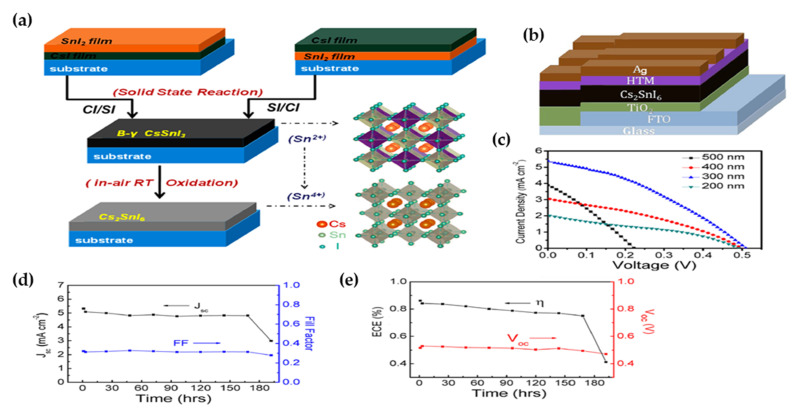
(**a**) Sketch of Cs_2_SnI_6_ film growth from CsSnI_3_ using wo-step deposition method through solid-state reaction; (**b**) sketch of the architecture for the Cs_2_SnI_6_ based perovskite solar cell; (**c**) J-V curves of perovskite solar cells fabricated with Cs_2_SnI_6_ of different thicknesses; (**d**,**e**) Cs_2_SnI_6_-based unsealed perovskite solar cell stability measurement of the main parameters as a function of time taken at specific time intervals. Reproduced with permission from [86]. Copyright 2017, Elsevier.

In 2017, Qui et al. [86], as already provided in Table 2, by developing a two-step sequential deposition method, prepared B-γ-CsSnI_3_ thin film for PV application. However, under the ambient environment the oxidation of Sn changed from 2+ to 4+, which resulted in vacancy-ordered air-stable Cs_2_SnI_6_ perovskite with a bandgap of 1.48 eV. The best power conversion efficiency of 0.96% was measured for PSC with perovskite film of 300 nm thickness, exhibiting the V_oc_ of 0.51 V, J_sc_ of 5.41 mA cm^−2^ and FF of 35. The inefficient electron/hole extraction towards the electrodes was explained by the mismatched energy-barrier between TiO_2_/Perovskite/HTL layers resulting in poor efficiency of ~1% as shown in Figure 24a–c.

A Ti-based perovskite solar cell was fabricated first by Chen et al. [87]. The insertion of C_60_ between TiO_2_-ETL layer and Cs_2_TiBr_6_ resulted in promising efficiency of 3.3% with V_oc_ of 1.02 V, J_sc_ of 5.69 mA cm^−2^ and FF of 0.564 in a reverse scan.

## 3. Conclusions and Perspective

Despite the significant power conversion efficiency of Pb-based perovskites for PV applications, two main drawbacks including the instability and toxicity of Pb hinder their large-scale fabrication and commercialization. To develop non-toxic and air-stable photo- absorbers for PSCs, double halide perovskites with a formula of A_2_M^+^M^3+^X_6_ were suggested and investigated as a new design strategy.

In this review, theoretical and experimental results of Cs-based Pb-free double halide perovskites, influence of metal doping/alloying on selected perovskites along with current potential applications are highlighted. A thorough review of Cs_2_M^+^M^3+^X_6_ structures with special emphasis on (Bi^3+^, In^3+^, Sb^3+^) as M^3+^ elements is carried out. In addition, Cs-based vacancy ordered double halide perovskites with formula of Cs_2_M^4+^X_6_ are also reviewed.

Based on the obtained results from different research methodologies and procedures, it is clear that Cs-based Pb-free double halide perovskites have enhanced thermal and ambient stability, but the bandgap values of these materials are not optimal for PV applications. However, many studies have demonstrated that bandgap of these materials could be tuned through metal doping/alloying.

Among the various studied Cs_2_M^+^M^3+^X_6_ structures, Bi^3+^-based double perovskites have shown promising features for PV applications with an estimated indirect bandgap varying from ≈1.7 eV [56] to 2.89 eV [59] which could be tuned from indirect to direct by metal doping [91]. Also, to the best of our knowledge, the latest study based on Cs_2_AgBiBr_6_ showed the best photovoltaic efficiency of 3.11% [66] with unique features such as high environmental stability, low toxicity and long carrier recombination lifetime. Moreover, significant optical and electronic properties with remarkable performances, turn the Cs_2_AgBiBr_6_ into an outstanding candidate for other optoelectronic applications such as LEDs, UV detectors, X-ray detectors etc. Processes related to employing appropriate coating methods and managing bulk engineering of Cs_2_AgBiBr_6_ structure, along with managing interface engineering by for example introducing interfacial layers between ETL/Cs_2_AgBiBr_6_/HTL have to be concentrated for further enhancement of efficiency of PSCs based on this promising material.

Studies have revealed that In^3+^-based double perovskites have large direct bandgaps varying from ≈3.23 eV to 3.57 eV [85,124]. It was indicated that the parity-induced forbidden transition in Cs/In^3+^-based double halide perovskites causes the optical transition obstruction and makes these materials unsuitable for solar devices, but these materials showed inherent environmental stability against air/moisture. Also, it was shown through alloying of metal ions, not only the PL properties of Cs/In- based double halide perovskites were significantly enhanced, an important feature for LEDs, but also the large direct band gap of this compound was tuned from ~3.5 eV to 2.54 eV [125].

The Cs/Sb^3+^ double halide perovskites were shown to have large indirect bandgap varying from ≈1.64 eV (single crystals) to 3.0 eV [62,68], that hinders them of showing desirable PV performance. Cs/Sb^3+^ double halide perovskites are thermodynamically unstable. Amongst vacancy-ordered lead-free double halide perovskites reviewed, Cs_2_TiBr_6_ showed the relatively higher efficiency of 3.3% [87] along with high stability under ambient environment.

Our review shows that by employing different theoretical and experimental approaches in the studies has had a significant influence on the results which describe optoelectronic characteristics of Cs-based double halide perovskites. For example, it was shown that synthetic methodologies and conditions (temperature, the concentration of precursors, time, solvents) and employing different types of characterization instruments (UV-Vis spectroscopy vs. DRS) led to obtaining varying photophysical and structural results, so in order to reduce/eliminate these discrepancies, as this review clearly indicates, a combined theoretical and experimental approach will be the best way to get in-depth understanding of lead-free double halide perovskite materials. It also helps to address the issues like bandgap alignment, carrier and interfacial dynamics between perovskite and transporting layers.

Furthermore, plenty of studies in order to employ suitable electrodes, HTL and ETL layers along with efficient interfacial layers could be carried out since these layers have a significant effect on PSCs′ efficiency. Exploring more on Cs-based double perovskite materials especially on Cs/Bi^3+^-based lead-free double halide perovskites may help the researchers to overcome the toxicity and instability challenges faced with lead-based perovskite materials for different applications in PV, photocatalysis, photodetectors, light-emitting devices etc.

## Figures and Tables

**Table 1:** Summary of prepared double halide perovskite samples based on inorganic Cs/Bi^3+^.

Compounds	Morphology	Synthetic Method	Optical Transition	Theoretical Bandgap	Experimental Bandgap	Characterization Techniques	Theoretical Calculation	Reference
Cs_2_AgBiBr_6_	Powder and single crystals	Solution-based process using hydrohalic acid	Indirect	-	1.95 eV	PL, UV-Vis spectroscopy, TGA, PXRD	-	[48]
Cs_2_AgBiBr_6_	polycrystalline	Solution-based process using hydrohalic acid process and solid-state reaction	Indirect	2.06 eV	2.19 eV	PL, UV-vis spectroscopy, XRPD	DFT-VASP	[49]
Cs_2_AgBiBr_6_	Single crystals	Solution-based process using hydrohalic acid with high pressure treatment	Indirect	2.36 eV	~1.7 eV	UV-Vis spectroscopy, ADXRD, Raman	DFT-LDA in CASTEP	[56]
Cs_2_AgBiBr_6_	Single crystals	Solution-based process using hydrohalic acid process and solid-state reaction	Indirect	1.8 eV	1.9 eV	PXRD, UV-Vis spectroscopy, PL	DFT-LDA+SOC, GW	[57]
Cs_2_AgBiBr_6_	-	-	Pseudo- direct	0.44 eV	-	-	DFT/VASP/PAW pseudopotentials/PBEsol, HSE06+SOC	[58]
Cs_2_AgBiBr_6_	Nanocrystals	Hot-injection method	Indirect	-	2.33 eV	TEM, HTEM, XRD, UV-Vis, PL	-	[59]
Cs_2_AgBiBr_6_	Single crystals and thin film	Solution-based process using hydrohalic acid, slow precipitation method	Indirect	-	2.0 eV	TRPL, PL, PDS, UV-Vis spectroscopy, X-ray photoemission	-	[60]
Cs_2_AgBiBr_6_	Single crystals	Solution-based process using hydrohalic acid	Indirect	2.1 eV	2.12 eV	Raman, PLE	DFT/Crystal17	[61]
Cs_2_AgBiBr_6_	Thin film	Solution-based process	Indirect	1.84 eV	2.10 eV	UV-Vis spectroscopy	DFT/PAW/VASP, HSE06+SOC, QTAIM/CRITIC2	[62]
Cs_2_AgBiBr_6_	Polycrystals	Mechanochemical	Indirect	-	2.0 eV	XRD, XPS, UV-Vis spectroscopy, TG-DSC, TCSPC, XRF	-	[63]
Cs_2_AgBiBr_6_	Thin film	Solution-based process using DMSO	Indirect	-	2.12 eV	XRD, UV-Vis spectroscopy, PL, SEM	-	[64]
Cs_2_AgBiBr_6_	Thin film	Solution-based process using DMSO	Indirect	-	2.02 eV	XRD, UV-Vis spectroscopy, PL, SEM, EDX, XPS, AFM	-	[65]
Cs_2_AgBiBr_6_	Thin film	Solution-based process using DMSO	Indirect	-	2.09 eV	XRD, UV-Vis spectroscopy, PL, SEM	-	[66]
Cs_2_InBiBr_6_	Powder and single crystals	solid-state reaction	Direct	0.33 eV	-	PXRD, SCXRD	DFT-VASP, PBE+SOC	[67]
Cs_2_AgBiCl_6_	Polycrystalline	Solution-based process using hydrohalic acid process and solid-state reaction	Indirect	2.62 eV	2.77 eV	PL, UV-vis spectroscopy, XRPD	DFT-VASP	[49]
Cs_2_AgBiCl_6_	Powder	Solid-state reaction	Indirect	3.0 eV	2.2 eV	PXRD, UV-Vis spectroscopy, PL	DFT-LDA Hybrid PBE0	[50]
Cs_2_AgBiCl_6_	Single crystals	Solution-based process using hydrohalic acid process and solid-state reaction	Indirect	2.4 eV	2.2 eV	PXRD, UV-Vis spectroscopy, PL	DFT-LDA+SOC, GW	[57]
Cs_2_AgBiCl_6_	Nanocrystals	Hot-injection method	Indirect	-	2.89 eV	TEM, HTEM, XRD, UV-Vis spectroscopy, PL	-	[59]
Cs_2_AgBiCl_6_	Single crystals and thin film	Solution-based process using hydrohalic acid, slow precipitation method	Indirect	-	2.5 eV	TRPL, PL, PDS, UV-Vis spectroscopy, X-ray photoemission	-	[60]
Cs_2_InBiCl_6_	Powder and single crystals	solid-state reaction	Direct	0.88 eV	-	PXRD, SCXRD	DFT-VASP, PBE+SOC	[67]
Cs_2_InBiCl_6_	-	-	Direct	1.02 eV	-	-	DFT, HSE+SOC, PBE+SOC	[54]
Cs_4_CdBi_2_Cl_12_	Single crystals	Solvothermal	Direct forbidden	3.58 eV	3.23 eV	PXRD, SC-XRD, TGA, EDS, PL, PLE, TRPL, UV-Vis-NIR spectroscopy	DFT/VASP/PAW, GGA-PBE, HSE+SOC	[68]

**Table 2:** Prepared device architecture and photovoltaic parameters of Cs-based double halide perovskite solar cells.

Perovskite Material	Year	Device Architecture	Perovskite Deposition Method	V_oc_ [V]	J_sc_ [mA cm^−2^]	FF	PCE [%]	Reference
Cs_2_AgBiBr_6_	2017	FTO/c-TiO_2_/mp-TiO_2_/perovskite/spiro-OMeTAD/Au	One-step spin coating with preheating	0.98	3.93	0.63	2.43	[77]
Cs_2_AgBiBr_6_	2018	ITO/SnO_2_/perovskite/P3HT/Au	One-step spin coating with low-pressure assisted method	1.04	1.78	0.78	1.44	[78]
Cs_2_AgBiBr_6_	2018	ITO/ c-TiO_2_/perovskite/spiro-OMeTAD/Au	One-step spin coating	1.06	1.55	0.74	1.22	[79]
Cs_2_AgBiBr_6_	2018	FTO/c-TiO_2_/mp-TiO_2_/perovskite/PTAA/Au	One-step spin coating with anti-solvent dropping methodology	1.02	1.84	0.67	1.26	[80]
Cs_2_AgBiBr_6_	2018	ITO/Cu-NiO/perovskite/C60/BCP/Ag	One-step spin coating with anti-solvent dropping methodology and post-annealing	1.01	3.19	0.69	2.23	[81]
Cs_2_NaBiI_6_	2018	FTO/c-TiO_2_/perovskite/P3HT/Au	One-step spin coating	0.47	1.99	0.44	0.42	[75]
Cs_2_AgBiBr_6_	2018	FTO/c-TiO_2_/mp-TiO_2_/perovskite/ spiro-OMeTAD/Au	Sequential vapor deposition with two step annealing	1.12	1.79	-	1.37	[82]
Cs_2_AgBiBr_6_	2019	FTO/TiO_2_/perovskite/SpiroOMeTAD/MoO_3_/Ag	Vacuum sublimation and one-step spin coating	1.01	3.82	0.65	2.51	[83]
Cs_2_AgBiBr_6_	2020	ITO/SnO_2_/perovskite/Zn-Chl /Ag	One-step spin coating	0.99	3.83	0.736	2.79	[64]
Cs_2_AgBiBr_6_	2020	FTO/c-TiO_2_/mp-TiO_2_/perovskite/ N719/spiro-OMeTAD/Au	One-step spin coating with two-step heating process.	1.06	5.13	0.524	2.84	[65]
Cs_2_AgBiBr_6_	2021	FTO/c-TiO_2_/mp-TiO_2_/C-Chl/perovskite/spiro-OMeTAD/Au	One-step spin coating	1.04	4.09	0.73	3.11	[66]
(Cs_1-x_Rb_x_)_2_AgBiBr_6_	2019	ITO/SnO_2_/perovskite/spiro-OMeTAD/Au	One-step spin coating with low-pressure assisted method	0.99	1.93	0.78	1.52	[84]
Cs_2_AgSbBr_6_	2019	FTO/c-TiO_2_/mp-TiO_2_/perovskite/spiro-OMeTAD/Au	One-step spin coating	0.35	0.080	0.35	0.01	[85]
Cs_2_SnI_6_	2017	FTO/c-TiO_2_/perovskite/P3HT/Ag	Two-step sequential vapor deposition	0.51	5.41	0.35	0.96	[86]
Cs_2_TiBr_6_	2018	FTO/TiO_2_/C60/perovskite/P3HT/Au	Facile low-temperature vapor deposition	1.02	5.69	0.564	3.3	[87]

**Table 3:** Summary of prepared doped- Cs-based double halide perovskite samples.

Compound	Dopant	Morphology	Synthetic Method	Characterization Techniques	Reference
Cs_2_(Ag_1–a_Bi_1–b_) Tl_x_Br_6_	Tl^+^ (0.003 < x < 0.075)	Polycrystalline Powder and single crystals	Solution-based process using hydrohalic acid	TRMC, ICP, Raman, SSRS, XPS, XAS, XANES, SC-XRD,	[91]
Cs_2_Ag (Bi_1−x_M_x_) Br_6_(M = In, Sb)	Sb^3+^ (x = 0, 0.125, and 0.375) In^3+^ (x = 0, 0.25, 0.5, and 0.75)	Powder	Solid-state reaction	PXRD, PL, UV-Vis spectroscopy	[92]
Cs_2_AgIn_x_Bi_1–x_Cl_6_	In^3+^ (x = 0, 0.25, 0.5, 0.75, and 0.9)	Nanocrystals	Anti-solvent recrystallization	XRD, TEM, PL, PLQE, HRTEM, TRPL, TCSPC, TA, XPS	[93]
Cs_2_Na_1−x_Bi_1−x_Mn_2x_Cl_6_	Mn^2+^ (x = 0, 0.002, 0.004, 0.012, 0.036)	Polycrystalline	Solution precipitation	PXRD, UV-Vis spectroscopy, EPR, PLQY, TGA, ICP-OES	[94]
(Cs_1−x_Rb_x_)_2_AgBiBr_6_	CsBr:RbBr at 100:0, 95:5, 90:10 and 85:15	Thin film	Solution-based process	UV-Vis spectroscopy, XRD, PL, IPEC, TRPL, SEM	[84]
Cs_2_NaBiCl_6_: Ag^+^, Mn^2+^, and Eu^3+^	Ag^+^, Mn^2+^, and Eu^3+^	Nanocrystals	Modified hot-injection method	PXRD, TEM, HRTEM, HAADF-STEM, EDS, XPS, UV-Vis spectroscopy, PLQY, ICP-OES,	[95]
Cs_2_AgInCl_6_: Mn^2+^	Mn^2+^(x = 0%, 0.1%, 0.3%, 0.9%)	Powder	Solution-based process using hydrohalic acid	ICP-AES, FESEM, XRD, TGA, PL, EPR, UV-Vis.spectroscopy,	[96]
Cs_2_AgInCl_6_: Mn^2+^	Mn^2+^ (x = 0.5% and 1.5%)	Nanocrystals	Colloidal hot-injection method	PL, PLE, PLQY, TRPL, ICP-OES, UV-Vis spectroscopy, TEM, HRTEM, XRD, XPS, ESR, DTA/TG GC-MS	[97]
Cs_2_Ag_1−x_Na_x_In_1−y_Bi_y_Cl_6_	Bi^3+^ and Na^1+^ (x = 0–1, y = 0.03–0.16)	Nanocrystals	Room temperature recrystallization process	UV-Vis spectroscopy, TEM, HRTEM, XRD, ICP-OES, PLQY, PL, SEM-EDS,	[98]
Cs_2_AgInCl_6_: Bi^3+^	Bi^3+^ (x = 0.1%)	Nanocrystals	Facile hot-injection method	XRD, EDS, TEM, HRTEM, UV-Vis spectroscopy, PL, PLE, PLQY	[99]
Cs_2_AgInCl_6_: Yb^3+^	Yb^3+^ (x = 0.1 to 1.6%)	Microcrystals and Colloidal nanocrystals	Precipitation method	ICP−AES, ICP−MS, XRD, FESEM, TEM, HRTEM, PL, PLE, TRPL, UV-Vis spectroscopy, TGA,	[100]
Cs_2_NaInCl_6_: Sb^3+^	Sb^3+^/In^3+^ (x = 0, 5.0%, 10%, 20%)	Powder	precipitation from an HCl solution	PXRD, TGA, UV-Vis spectroscopy, PLQY, PL,	[101]
Cs_2_NaInCl_6_: Sb^3+^	(Sb/(Sb+In) (x = 0, 5.0%, 10%, 15%, 30%, 60%, 100%)	Powder	Hydrotermal	XRD, UV-Vis spectroscopy, PL, TEM, XPS, PLE, HRTEM, Raman	[102]
Cs_2_Sb_1−a_Ag_1−b_Cu_2x_Cl_6_	Cu^2+^ (a + b = 2x, x = 0.00 (i.e., parent compound), 0.01, 0.05, and 0.10)	Polycrytalline	precipitation from an HCl solution	PXRD, EPR, NMR, ICP-OES, TGA, FESEM	[103]
Cs_2_AgSb_1−x_Bi_x_Cl_6_	Bi^3+^ (0 ≤ x ≤ 1)	Nanocrytals	Modified hot-injection method	XRD, FESEM, EDS, TEM, HRTEM, PL, PLE, UV-Vis spectroscopy,	[104]
Cs_2_AgSb_1−*y*_Bi*_y_*X_6_ (X = Br, Cl)	Bi^3+^ (0 ≤ y ≤ 1)	Nanocrytals	Modified hot-injection method	XRD, TEM, PL, Raman, TA, steady-state absorption	[105]
Cs_2_Sn_1−x_Te_x_I	Te^4+^(0 ≤ x ≤ 1)	Powder	Solution-phase synthesis	PXRD, UV-Vis spectroscopy, PL, XPS	[106]
Cs_2_SnCl_6_: Bi	Bi/(Sn + Bi) x = 0%, 0.99%, 4.76%, 9.09%, 13.04%, 16.66%, and 23.08%	Single crystals	Hydrothermal	XRD, ICP-OES, XPS, TGA, UV-Vis spectroscopy, PL, PLQY, PLE, TRPL	[107]
Cs_2_Sn_(1−x)_Ge_x_I_6_	Ge^4+^ (0 ≤ x ≤ 1)	-	-		[108]

**Table 4:** Summary of prepared double halide perovskite samples based on Cs/In^3+^.

Compounds	Morphology	Synthetic Method	Optical Transition	Theoretical Bandgap	Experimental Bandgap	Characterization Techniques	Theoretical Calculation	Reference
Cs_2_AgInBr_6_	-	-	Direct	1.50 eV	-	-	DFT/DFT/PBE/HSE	[119]
Cs_2_AgInCl_6_	Powder	Solution-based process using hydrohalic acid	Direct	2.1 ≤ E_g_ ≤ 3.3 (depends on calculation method)	3.3 eV	X-ray, UV-Vis spectroscopy, PL, TRPL,	DFT/LDA/HSE/PBE0/HSE	[118]
Cs_2_AgInCl_6_	Polycrystalline and single crystals	solid-state reaction and hydrothermal reaction	Direct	5.0 eV (without SOC)	3.53 eV	UV-Vis spectroscopy, SC-XRD, PXRD,	DFT-LDA, FP-LAPW+LO, SOC	[125]
Cs_2_AgInCl_6_	Nanocrystals	Hot-injection method	Direct	-	3.57 eV	STEM-EDS, UV-Vis-NIR spectroscopy, PL, PLE, TEM, XRD	DFT-VASP, PBE-GGA	[85]
Cs_2_AgInCl_6_	Powder	Hydrothermal method	Direct	3.33 eV	3.23 eV	TRPL, UV-Vis, XRD, SEM, UV-Vis spectroscopy, PL, TGA	DFT/PAW/PBE/VASP	[124]
Rb_2_AgInBr_6_	-	-	Direct	1.46 eV	-	-	DFT/DFT/PBE/HSE	[119]
Rb_2_CuInCl_6_	-	-	Direct	1.36 eV	-	-	DFT/DFT/PBE/HSE	[119]

**Table 5:** Summary of prepared double halide perovskite samples based on Cs/Sb^3+^.

Compound	Morphology	Synthetic Method	Optical Transition	Theoretical Bandgap	Experimental Bandgap	Characterization Techniques	Theoretical Calculation	Reference
Cs_2_AgSbBr_6_	Single crystals and thin film	Hydrothermal and Solution-based process using hydrohalic acid	Indirect	1.46 eV	1.64 eV for crystals 1.89 eV for mixed-phase film	XRD, XPS, UV-Vis spectroscopy, XRF	DFT/PAW/VASP, HSE06+SOC, QTAIM/ CRITIC2	[62]
Cs_2_AgSbBr_6_	Polycrystalline	Mechanochemical	Indirect	-	1.93 eV	XRD, XPS, UV-Vis spectroscopy, TG-DSC, TCSPC, XRF	-	[63]
Cs_2_AgSbCl_6_	Polycrystalline and single crystals	Solid-state reaction and hydrothermal reaction	Indirect	2.4 eV	2.54 eV	UV-Vis spectroscopy, SC-XRD, PXRD,	DFT-LDA, FP-LAPW+LO, SOC	[125]
Cs_2_AgSbCl_6_	Crystals	Hydrothermal	Indirect	2.35 eV (by HSE) 1.40 eV (by PBE)	2.24 eV ≤ E_g_ ≤ 2.61 eV (depends on HCl amount)	XRD, UV-Vis-NIR spectroscopy, SEM, TG-DSC	DFT-VASP, PAW, GGA-PBE, HSE	[124]
Cs_2_AgSbCl_6_	Nanocrystals	Hot-injection method	Indirect	-	2.53 eV without AgCl 2.57 eV with AgCl	STEM-EDS, UV-Vis-NIR spectroscopy, PL, PLE, TEM, XRD,	DFT-VASP, PBE-GGA	[85]
Cs_4_CuSb_2_Cl_12_	Polycrystalline	Solution-based process using hydrohalic acid	Direct	0.98 eV by Dmol^3^ 1.63 eV by CASTEP 2.44 eV by VASP	1.02 eV	PXRD, TGA, UV-Vis-NIR spectroscopy, UV stability test, SC-XRD	DFT/Dmol3/CASTEP/VASP	[129]
Cs_4_CdSb_2_Cl_12_	Single crystals	Solvothermal	Direct forbidden	3.29 eV	3.0 eV	PXRD, SC-XRD, TGA, EDS, PL, PLE, TRPL, UV-Vis-NIR spectroscopy	DFT/VASP/PAW, GGA-PBE, HSE+SOC	[68]

**Table 6:** Summary of prepared vacancy-ordered double halide perovskite samples.

Compound	Morphology	Synthetic Method	Optical Transition	Theoretical Bandgap	Experimental Bandgap	Characterization Techniques	Theoretical Calculation	Reference
Rb_2_SnI_6_	Powder	Solution precipitation	Direct	1.13 eV and 1.32 eV (depend on the calculation)	1.32 eV	XPDF, nPDF, UV-Vis spectroscopy, PPMS, SXRD, PXRD	DFT-VASP, PAW, PBEsol, HSE06, HSE06+SOC, DFPT	[106]
Cs_2_AuI_6_	-	-	Direct	1.31 eV	-	-	DFT-VASP, PAW, PBE, HSE	[138]
Cs_2_TiBr_6_	Powder	melt-crystallization method	Indirect	2.01 eV (HSE) 1.89 eV (HSE+SOC)	1.78 eV	UV-Vis spectroscopy, XRD	DFT-VASP, PAW, HSE06	[139]
Cs_2_TiI_2_Br_4_	Powder	melt-crystallization method	Indirect	1.49 eV (HSE) 1.32 ev (HSE+SOC)	1.38 eV	UV-Vis spectroscopy, XRD	DFT-VASP, PAW, HSE06	[139]
Cs_2_TiI_6_	Powder	melt-crystallization method	Indirect	1.20 eV (HSE) 1.05 eV (HSE+SOC	1.02 eV	UV-Vis spectroscopy, XRD	DFT-VASP, PAW, HSE06	[139]
Cs_2_TiI_4_Br_2_	Powder	melt-crystallization method	Indirect	1.10 eV (HSE) 1.00 eV (HSE+SOC	1.15 eV	UV-Vis spectroscopy, XRD	DFT-VASP, PAW, HSE06	[139]
Cs_2_Ti (Br*_x_*Cl_1−*x*_)_6_ (0 < x < 1)	Powder	Solution-based process using hydrohalic acid	Quasi-direct	∼1.6 eV to ∼2.3 eV	∼1.7 eV to ∼2.5 eV	SEM, EDS, XRD, TGA, UV-Vis-NIR spectroscopy, PL, PLQY	DFT-CASTEP, GGA-PBE	[141]

## Data Availability

No new data were created or analyzed in this study. Data sharing is not applicable to this article.
